# A scoping review of robustness concepts for machine learning in healthcare

**DOI:** 10.1038/s41746-024-01420-1

**Published:** 2025-01-17

**Authors:** Alan Balendran, Céline Beji, Florie Bouvier, Ottavio Khalifa, Theodoros Evgeniou, Philippe Ravaud, Raphaël Porcher

**Affiliations:** 1 Université Paris Cité, Université Sorbonne Paris Nord, INSERM, INRAE, Centre for Research in Epidemiology and StatisticS (CRESS), Paris, France; 2 INSEAD Decision Sciences, Fontainebleau, France; 3 Centre d’Épidémiologie Clinique, Assistance Publique-Hôpitaux de Paris, Hôtel-Dieu, Paris, France; 4 Columbia University Mailman School of Public Health, Department of Epidemiology, New York, USA

**Keywords:** Classification and taxonomy, Machine learning

## Abstract

While machine learning (ML)-based solutions—often referred to as artificial intelligence (AI) solutions—have demonstrated comparable or superior performance to human experts across various healthcare applications, their vulnerability to perturbations and stability to variations due to new environments—essentially, their robustness—remains ambiguous and often overlooked. In this review, we aimed to identify the types of robustness addressed in the literature for ML models in healthcare. A total of 274 eligible records were retrieved from PubMed, Web of Science, IEEE Xplore, and additional sources. Eight general concepts of robustness emerged. Furthermore, an analysis of those concepts across types of data and types of predictive models revealed that the concepts were differently addressed. Our findings offer valuable insights for stakeholders seeking to understand and navigate the robustness of machine learning models during their development, validation, and deployment in healthcare settings, where interpretation of robustness may vary.

## Introduction

Solutions such as software and medical devices integrating artificial intelligence (AI) or machine learning (ML) have become common in healthcare^1^. They are no longer merely in the state of development but are gaining increasing attention for real-world applications, as evidenced by the growing number of FDA-approved medical devices using AI^2^.

AI-enabled medical devices for decision support, and more precisely the ML models within these medical devices, have shown comparable performance—and sometimes even better performance—than experts for different tasks^3–5^. However, they are usually not assessed prospectively or under actual clinical workflow conditions^6,7^. Hence, their apparent performance does not take into account external sources of perturbations and variations that can affect the model behaviour^8,9^. Moreover, some AI-based solutions are being designed for deployment outside traditional clinical settings, such as mobile devices like smartphones^10^. This shift raises concerns regarding the potential introduction of additional sources of perturbations.

Resilience of ML models to variations and perturbations due to the environment in which the model operates is commonly referred to as *robustness*^11^. Besides, robustness has also been identified as a key principle in trustworthy AI frameworks. For example, the ALTAI framework considers robustness as one of the three core components of trustworthy AI^12^. Similarly, the FUTURE-AI framework that proposed a guideline for trustworthy AI in healthcare, considers robustness as an essential principle—on par with fairness and explainability—in achieving trustworthy AI in healthcare^13^. This highlights that robustness is not just a property of a machine learning model but a fundamental principle toward the implementation of safe and trustworthy AI in healthcare, and beyond.

However, the practical implications of this term remain somewhat ambiguous. Robustness serves as an overarching concept encompassing various factors that can impact a model differently, depending on the nature of perturbations but also on the development stage of the machine learning model (e.g. acquiring data, selecting and training a machine learning model, and evaluating it on new data). Moreover, different types of perturbation can have varying impacts on the performance of ML models. For instance, adversarial attacks—a method to inject noise in an image while preserving visual information—have demonstrated a remarkable ability to fool deep learning models’ prediction^8^. Such blindspots could confuse and raise legitimate concerns in healthcare, where ML models, especially those based on deep learning, considered black-box due to their limited interpretability, are employed for critical tasks such as diagnosis, prognosis, or patient monitoring^14^.

However, it is important to note that the use of “black-box” solutions for managing patient health is neither new nor exclusive to algorithms. A complete understanding of a drug’s effects, especially its adverse effects, cannot be fully achieved through clinical trials alone. Rare or unexpected adverse effects can emerge due to deviations in the target population and drug administration when used in real-life settings. Furthermore, such effects can also occur within the same target population used during clinical trials when a drug is used on a larger scale. Cases like Thalidomide show that clinical trials are insufficient to capture all adverse events in specific populations, particularly due to ethical concerns and unobserved outcomes during clinical trials, such as effects on newborns. Similarly, machine learning and deep learning models, and by extension, the medical devices that incorporate them, may exhibit unexpected behaviours when applied to populations or contexts beyond those they were originally trained and tested on. This raises the critical question of how to ensure that those non-deterministic solutions do not deviate or behave unpredictably once deployed in real-world clinical practice. The Thalidomide scandal was a catalyst for the creation of pharmacovigilance, which now also sparks discussions about applying a similar monitoring framework for AI-based devices in healthcare—often referred to as algorithmovigilance^15^.

Moreover, the development and deployment of ML models in healthcare involve multiple stakeholders, each with their concerns and perceptions regarding what constitutes a variation or perturbation for these models. Different stakeholders, including researchers, model developers, operators, healthcare professionals, and patients perceive and encounter different types of variations. Given these diverse perspectives, the current understanding of robustness as simply handling “variations and perturbations” is inadequate.

Therefore, a clear and comprehensive view of the concepts of robustness is essential to ensure effective communication among all stakeholders in healthcare about what a model is robust against. By identifying the specific causes of perturbations that can occur throughout the life cycle of an ML-based solution in healthcare, it becomes possible to address these issues and mitigate their impact on the model’s performance. Given the need to clarify the concept of machine learning robustness and identify the key characteristics related to that concept, we decided to conduct a scoping review^16–18^. Namely, the objectives of this scoping review were (1) to identify the various concepts of robustness currently used in the literature for machine learning models in healthcare for decision support; and (2) to map those different concepts of robustness across types of data and of predictive models.

## Results

### General characteristics

**Fig. 1:**
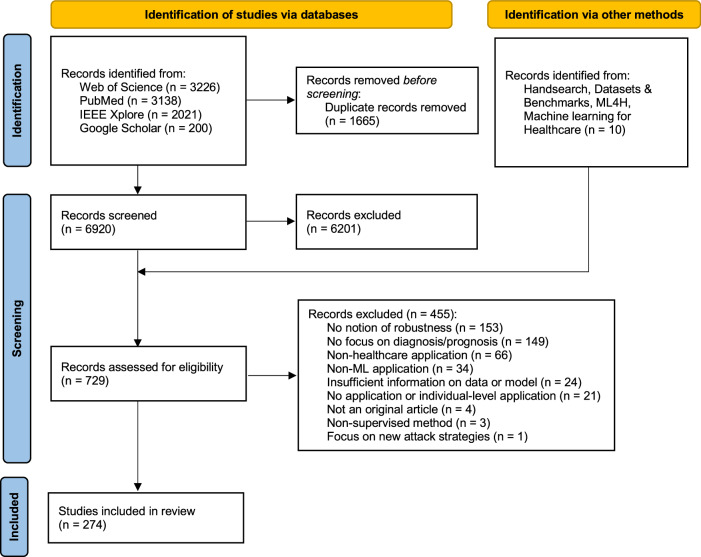
PRISMA flow diagram. The flow chart indicates the different number of records kept and excluded at each step of the screening process.

The search initially retrieved 8585 records, of which 6920 remained after removing duplicates. Subsequently, 6201 records were excluded based on title and abstract screening. Ten were added through other methods. After assessing the full text of 729 records, 274 were finally included in our review. Figure 1 provides a summarised overview of the screening process. The list of included studies is available in Supplementary Table 1. Among the included records, 190 (69%) were published in journals, while 81 (30%) originated from conferences, workshops, and symposiums. One record each was from a scholarly dissertation, a preprint, and a book chapter. From these 274 records, we extracted 526 combinations of medical specialties, model type, data type, and concepts of robustness, presented in the following section—all summarised in Table 1.

**Table 1:** General characteristics of included studies

	Combinations (*N* = 526)
**Medical discipline**	
Pulmonology	86 (16.3%)
Gynaecology	85 (16.2%)
Neurology	73 (13.9%)
Dermatology	33 (6.3%)
Gastroenterology	32 (6.1%)
Intensive care medicine	32 (6.1%)
Ophthalmology	29 (5.5%)
Cardiology	23 (4.4%)
Urology	22 (4.2%)
Haematology	21 (4.0%)
Neuro-oncology	20 (3.8%)
Psychiatry	15 (2.9%)
Infectious diseases	11 (2.1%)
Endocrinology	9 (1.7%)
ENT	7 (1.3%)
Other	28 (5.3%)
**Data type**	
Image	167 (31.7%)
Omics	105 (20.0%)
Image derived features	84 (16.0%)
Clinical data	82 (15.6%)
Physiological signal	41 (7.8%)
Multimodal	14 (2.7%)
Other	33 (6.3%)
**Model type**	
Deep learning	269 (51.1%)
Non-deep machine learning	134 (25.5%)
Linear regression model	64 (12.2%)
Hybrid model	15 (2.9%)
Other	44 (8.4%)

ENT: Ear-nose-throat.

The five most frequent domains of applications were *pulmonology* (86/526, 16.3%), followed by *gynaecology* (85/526, 16.2%), *neurology* (73/526, 13.9%), *dermatology* (33/526, 6.3%), and *gastroenterology* (32/526, 6.1%).

The most common type of data used to build predictive models was *image data* (167/526, 31.7%), followed by *omics data* (105/526, 20%), and *image-derived features* (84/526, 16%). The latter corresponds to cases where features are derived from images before training the predictive models, as opposed to end-to-end learning using images directly. The *other* category (47/526, 8.9%) encompassed other types of data such as vocal recordings, gait data, and clinical discharge summaries extracted from EHRs. A complete list of all items categorised as *other* for each extracted domain is presented in Supplementary Table 2.

The majority of the applications used *deep learning-*based methods (269/526, 51.1%), followed by *non-deep machine learning methods* (134/526, 25.5%). The latter includes methods such as decision tree, random forest, gradient boosting, and support vector machine. *Hybrid methods*, which combine deep learning (for feature extraction) and non-deep learning/linear methods for predictions, were present in only 15 out of the 526 applications (2.9%). Linear regression models accounted for 12.2% (64/526). *Other* types of predictive models (44/526, 8.4%) comprised models such as naive Bayes, Gaussian processes, and linear/quadratic discriminant analysis.

**Fig. 2:**
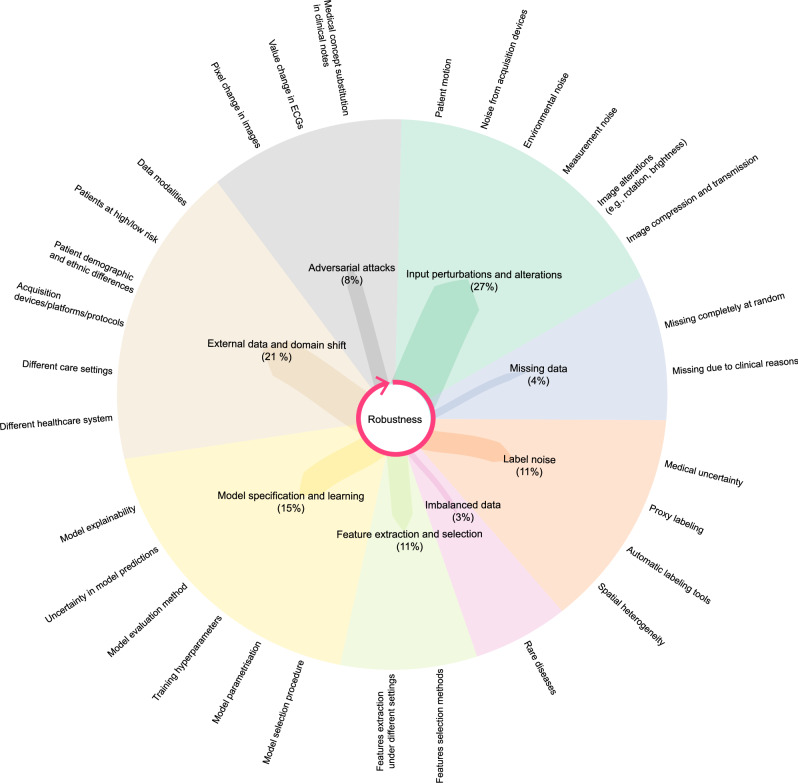
The eight robustness concepts. The radial dendrogram shows the distribution of the eight robustness concepts based on combinations of predictive models and data types from the included studies. Examples of different sources of variations for each concept are also provided. The arrow indicates the order in which these concepts can intervene during the lifecycle of a machine learning model.

Eight general concepts of robustness emerged, illustrated in Fig. 2 and described in Table 2: *input perturbations and alterations, missing data, label noise, imbalanced data, feature extraction and selection, model specification and learning, external data and domain shift*, and *adversarial attacks*. Examples of notions for each concept are described in Supplementary Table 3. The most frequently addressed concept was robustness to *input perturbations and alterations* (142/526, 27%), while robustness to *imbalanced data* (15/526, 3%) was the least commonly tackled. The metrics used to assess the robustness to the different concepts are described in Supplementary Table 4.

**Table 2:** Description of identified concepts with examples

Concept	Explanation	Examples
Input perturbations and alterations	Perturbations that can arise on the input during its acquisition, measurement, manipulation or pre-processing. Those perturbations are generally encountered or caused unintentionally (as opposed to adversarial attacks described below).	Robustness to Gaussian noise in continuous features^53^ Robustness to plausible alteration encountered in medical setting: horizontal and vertical translation/rotation/brightness variation/zoom/Gaussian noise/blur addition/JPEG compression^54^ Robustness to outliers in gene expression data^55^
Missing data	Part of the input is not available.	Robustness to the number of available inputs during test time and also to specific input subsets based on their clinical utility and their practicality in terms of cost and ease of use^31^ Robustness to random data missingness, non-random missingness, and complete feature missingness^56^
Label noise	Uncertainty in the labels used to train the predictive model.	Recorded treatment decisions used as labels instead of disease diagnosis^34^ Label noise from inconsistency among expert opinions^35^
Imbalanced data	Samples from one or more classes are significantly underrepresented compared to the others.	Robustness to data where the outcome of interest (death by COVID-19) is underrepresented (11%) compared to those who survived (89%)^57^
Feature selection and extraction	Variability in selected subset of features used to train the model and reliability of extracted features to different conditions.	Robustness of features identified by the model across different subset of the data^58^ Robustness to the feature selection method^59^ Robustness of extracted features (radiomics) to different contrast (illumination) settings in ultrasound imaging^60^
Model specification and learning	Variability resulting when selecting, parametrizing, training and interpreting a predictive model.	Robustness to hyperparameters tuning (stable performances over several hyperparameter values)^61^ Robustness of the model training strategy (pretraining, finetuning with/without freezing some layers, iterative training on multiple datasets, etc.)^62^
External data and domain shift	Model evaluation on different populations, tasks, data modalities, or care settings.	Robustness across care settings (admission types: elective procedures, urgent care, emergency, trauma centre), age groups, genders, and races^23^ Robustness to the presence of co-occurring conditions: allergic disorders, gastrointestinal (GI) disorders, immune metabolic (I/M) disorders, and neurological disorders^63^ Variations in CT protocols (scanner model, patient size, radiation dose, or reconstruction parameters)^64^ Robust across various acquisition views of MRI examinations performed before treatment: both axial and sagittal views^65^
Adversarial attacks	Input voluntarily altered to change the predictions of a predictive model.	Substitution of medical concepts, replacement of words, removal of adverbs on EHR clinical notes^30^ Adversarial attacks on diagnostic images for cancerous lesion classification^28^ Adversarial attacks on ECG signal classification^29^

### Robustness concepts across types of data and predictive model

**Fig. 3:**
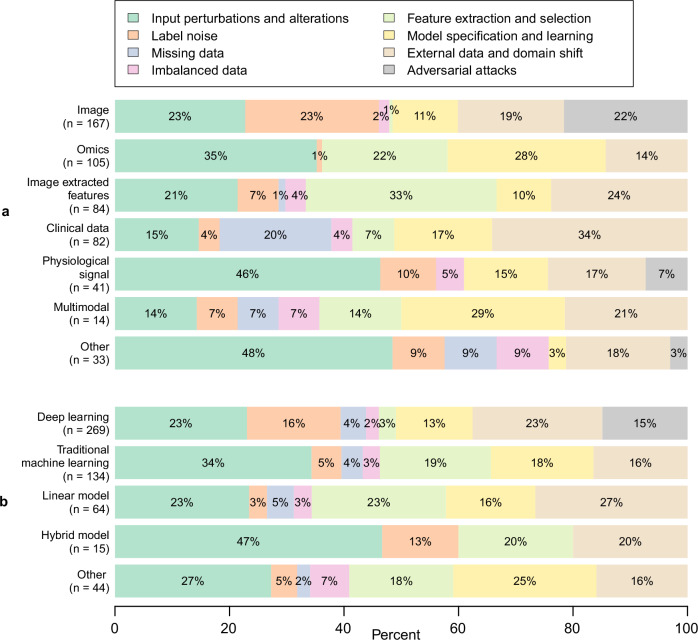
Stratified view of the eight concepts of robustness. The eight robustness concepts are stratified by data type **a** and predictive model type **b**. The emphasis on specific robustness concepts varies depending on the type of data or predictive model used. For instance, panel **a** shows that the *feature extraction and selection* concept is more commonly emphasized for high-dimensional tabular data, such as omics or image-derived features. Similarly, panel **b** shows that adversarial attacks are primarily addressed in deep learning-based applications.

We also analysed the robustness concepts based on the data and models used. The results, shown in Fig. 3, reveal that robustness concepts were addressed differently depending on the choice of data and model. We highlight some of these findings below.

Figure 3a illustrates the different concepts stratified by the type of data used to develop a model. Robustness to the concept of *feature extraction and selection* was mainly emphasised in applications based on *image-derived data* (33%) and *omics* (22%). *Adversarial attacks* were mainly tackled in applications relying on *image* (22%) and *physiological signal* data (7%). Applications addressing robustness to missing data were for the most part accounted for applications using *clinical data* (20%). Robustness to *label noise* was most frequently addressed in *image*-based applications (23%). Applications relying on *omics* data (i.e., data obtained through high-throughput measurement of biological molecules) addressed the fewest number of concepts of robustness (5).

Figure 3b illustrates the different concepts stratified by the type of the model. Robustness to *adversarial attacks* was only addressed for applications based on *deep learning* (15%). Robustness to *label noise* was also mostly tackled for *deep learning* models (16%) and *hybrid models* (13%). The concept of *external data and domain shift,* as well as *input perturbations and alterations,* were the most addressed concepts across all types of models. Applications based on *hybrid models* addressed the smallest number of concepts (4), while applications based on *deep learning* covered the most (8).

An analysis of robustness concepts, stratified by medical specialty, is presented in Supplementary Table 5.

Moreover, an assessment of the combination between the type of data and the type of predictive model, available in Supplementary Fig. 1, revealed that deep learning models were primarily favoured for images, while non-deep learning methods, such as non-deep machine learning and linear regression models, were largely used for omics data.

## Discussion

Building on the different notions extracted from the studies included in this scoping review, we identified eight general concepts to represent the robustness of machine learning models for different sources of perturbations: *input perturbations and alterations, missing data, label noise, imbalanced data, feature extraction and selection, model specification and learning, external data and domain shift*, and *adversarial attacks*. These concepts encompass perturbations and variations that can occur at different stages of a machine learning model’s life cycle. For example, input alterations and perturbations, missing data, label noise, and imbalanced data, occur during data acquisition, collection, and preparation; feature selection and extraction, model specification and learning are part of model development; and external data and domain shift, and adversarial attacks relate to model validation and deployment, as illustrated in Fig. 2. This classification highlights the diverse nature of robustness in ML solutions for healthcare applications.

The review also highlights the intersection between robustness and other intrinsic machine learning principles such as *generalizability*, *fairness*, and *explainability*. For instance, studies that evaluate the robustness to various data distributions through external validation inherently address the generalizability of the model^19–22^. Similarly, addressing the robustness of the model to specific minority groups directly relates to the notion of fairness^23–25^. Assessing the robustness of a model’s learned features to ensure the absence of spurious correlations is also intricately linked to model explainability^26,27^.

Our review also revealed that certain sources of perturbations were more subject to the choice of the predictive model or the type of data. For instance, adversarial attacks were used mostly (90%) to assess the robustness of deep learning models trained on image data. This is explained by the nature of adversarial attacks being perturbations that maintain the “visual” structure (from a human perspective) of the data as close as possible to the original data, which is more suited for image and ECG than for tabular data, for instance^28,29^. Only one study addressed adversarial attacks for clinical notes by applying transformations such as medical concept substitution, word replacement, or adverb removal^30^. Similarly, robustness to feature selection and extraction was mostly addressed for applications based on omics data (e.g. genomics, proteomics, metabolomics, etc.) and to image-derived data (e.g. radiomics) which are considered high-dimensional data. For applications based on such data, it is generally desirable to select only a subset of the most salient or most reliable features, introducing additional sources of perturbations. Robustness to missing data was mainly addressed for clinical data due to the inherent nature of missing data. Studies addressing missing data were not limited to a particular missingness mechanism (e.g., missing completely at random, missing at random, missing not at random)^31,32^. One study based on multimodality discussed robustness in settings where an entire modality was missing^33^. Furthermore, we observed that the choice of model is influenced by the type of data. For instance, deep learning models are favoured for image-based applications, while non-deep machine learning and linear regression models are often used for high-dimensional data, such as omics. This choice consequently impacts the robustness concepts that we retrieved from the literature.

Other concepts such as *input perturbations and noisy environments* and *label noise* are more general. This is particularly evident in the case of label noise, which can manifest in various forms depending on the dataset. Label noise can stem from training on proxy labels, for instance, deriving labels from treatment prescriptions^34^. Label noise can also derive from human uncertainty when using the consensus of multiple annotators, often needed due to the absence of a pathologically confirmed diagnosis label, thereby introducing inherent uncertainty^35–37^. For large data, it is often infeasible to perform the data labelling by human annotators, thus resorting to tools from natural language processing (NLP) to automatically infer labels for the data, creating noise in the process^38^. Moreover, discrepancies may also arise when a label assigned at an image level (e.g. a whole slide image (WSI) or a chest x-ray) fails to accurately capture the heterogeneity present locally in the image (e.g. in patches extracted from the WSI or specific areas of a chest x-ray)^39,40^.

Our classification also shows that these concepts can be perceived and associated with different stakeholders. If we put into perspective the different concepts of robustness identified in our review with what is known regarding the different actors involved in the different steps of a machine learning model lifecycle, we find that those concepts are associated with different stakeholders. For instance, patients’ perceptions of robustness mainly fall within the space of input perturbations and alterations that generally occur during data acquisition, which involves patients. Variations related to external data and domain shifts are more likely to be experienced by ML-system operators, patients, and healthcare professionals when the model is deployed and used in a setting for which it was not initially trained. Conversely, variations occurring during the machine learning model specification and learning are mainly observed by model developers. Adversarial attacks, although effective at exploiting deep learning models’ blind spots, are intentionally generated samples, usually by those developing or validating models. Thus, they are not encountered in real clinical settings, making them less associated with patients, healthcare professionals, or ML-system operators.

Nearly half (47%) applications reviewed focused on medical specialties within gynaecology, pulmonology, and neurology. This can be attributed to the availability of easily accessible benchmark datasets. For instance, the *ChestX-ray8* dataset, a publicly available dataset of 108,948 chest X-rays covering eight diseases^41^, contributes to the high number of applications in pulmonology. Similarly, the large number based on omics can be ascribed to The Cancer Genome Atlas Program (TCGA), a publicly available collection of genomes that was developed mainly for cancer-related research^42^. The main gaps in studies addressing the least-explored medical applications, predictive models, and type of data can be explained by the overall scarcity of machine learning research in these areas.

Our review has some limitations. First, while it provides valuable insights into the different dimensions of robustness for a machine learning model in healthcare, it is not meant to provide or assess methods for “robustifying” a model. This requires additional efforts and the implications of a multidisciplinary team involving healthcare professionals, model developers, and AI/ML researchers. For instance, perturbations caused by label noise can be addressed in many ways: at the data collection level by collecting additional data with more reliable ground truth labels or by correcting existing labelling errors before training the predictive model^43^. It could also be addressed from the model perspective, where strategies to mitigate the impact of label noise may include developing models that are inherently robust to such noise, or by adapting model architectures and training strategies to correct noisy labels during training^44,45^. Second, our review is mostly restricted to studies associated with the terminology ‘robust’, ‘noise, and ‘perturbation’. Our choice was motivated by the frequent use of these terms in the machine learning community and to limit the number of records obtained through the search. Other terms could have been chosen, such as 'stability', 'resilience', 'reliability', or 'vulnerability', which might have produced different studies. Third, our review is, by its nature, limited temporally. Indeed, our review reflects the evidence available in the literature at a given time. Therefore, it does not include more studies based on more recent machine learning methods such as foundation models, which have recently demonstrated impressive performance across various tasks. While these models are currently a hot topic, their use in healthcare—and particularly their robustness—remains in its early stages. A search (29th October 2024) using terms like ‘foundation model,’ ‘generalist medical artificial intelligence,’ and ‘artificial general intelligence’ returned only two eligible studies, based on the title and abstract, related to foundation models across the 3 databases, which were all published after the search date of this work. We believe that the robustness of foundation models will become a distinct area of study due to their unique characteristics (e.g., multi-modality, specific training strategy, finetuning, different evaluation methods, etc.).

Other works have focused on the robustness of machine learning models. A study published in 2020 explored various notions of robustness in healthcare but did not examine how these concepts varied based on the type of data and predictive model used^46^. Another study in 2023 proposed a general definition for robustness from a causal perspective and they did not focus on healthcare^47^. Moreover, both studies did not rely on a formalised review methodology. To our knowledge, this review presents the first classification of robustness concepts specifically tailored for machine learning in healthcare. It’s important to note that different choices in our approach could have resulted in a different classification^48^.

We believe that our review constitutes the first step toward identifying appropriate strategies and frameworks for each of the proposed concepts of robustness to improve the robustness of a machine learning model both during the development of the model but also after the model has been deployed where it is crucial to ensure that the model remains robust in time. Future work based on this study can take different directions: one focus could be on identifying the various methods developed to address and mitigate robustness issues in machine learning models for each of the concepts we identified. Another direction could involve stress testing different machine learning models against the concepts identified in this study to explore their robustness and determine which factors are more likely to impact model performance.

Given the discrepancy between the ideal environment in which a model is developed and the complex real-world setting characterised by numerous sources of perturbations and variations, establishing a robust framework to identify and mitigate these potential sources in healthcare is crucial. Our review provides a comprehensive overview of the different concepts of robustness that are addressed at various stages of an ML model’s life cycle in healthcare for decision support. The perception of what constitutes a perturbation or variation can differ based on the life cycle stage, type of data, predictive model, and the various stakeholders involved. Our review may help stakeholders navigate and comprehend the robustness of models deployed in healthcare settings, improving the reliability of these models in real-world applications.

## Methods

The proposed review was conducted and reported per the Joanna Briggs Institute methodology and Preferred Reporting Items for Systematic reviews and Meta-Analyses extension for Scoping Reviews (PRISMA-ScR) also detailed in Supplementary Table 6^49,50^.

A study protocol was uploaded on Open Science Framework^51^.

### Search strategy and selection criteria

Relevant studies for our review were identified in two ways. First, we searched three electronic databases: PubMed, IEEE Xplore, and Web of Science (search date: 1 March 2023). These three databases cover a broad range of machine learning literature in healthcare, making them suitable for our review. A search equation was developed with the assistance of a librarian (CS). Search terms such as “deep learning”, “machine learning” were selected to retrieve machine learning studies, as well as terms like “statistical learning” and “prediction model” to capture studies from the field of statistics. The detailed search equations for each different database can be found in Supplementary Table 7–9. We did not set a limit for the start date when searching for eligible studies.

Additional studies were also searched in the grey literature. We retrieved the first 200 records on Google Scholar obtained using the keywords “machine learning”, “robustness”, and “healthcare”, and we manually searched records available on relevant conferences and associated workshops websites. These conferences and workshops include *Datasets and Benchmarks*, *Machine learning for Health*, and *Machine learning for Healthcare*.

Additional studies identified in the reference lists of included studies were also considered.

We included a record if it described a notion of robustness for a supervised or semi-supervised machine learning model developed for healthcare applications related to decision support such as diagnosis, prognosis, or treatment recommendation. Thus, we included records both on classification and regression tasks.

Records about treatment recommendations were excluded if the notion tackled by the study was related to so-called “doubly-robust” methods^52^. Since the doubly-robust property is specific to estimators developed within a causal framework, we did not include them, as this robustness property would not be transposable to non-causal settings.

Records that used the term robust but for which the notion was vague or not well defined were also excluded.

We excluded records if information regarding either the predictive model used or the data used to develop the model was insufficient to allow data extraction.

Records focusing on adversarial attacks were excluded if the paper only proposed a novel method to design adversarial samples.

Only records written in English and with an abstract were considered.

### Study selection

Retrieved studies were imported to the Covidence software to remove duplicates and for screening.

Two reviewers (AB and CB) independently screened 200 randomly selected records. Any uncertainty during the title and abstract screening was discussed and resolved between the two reviewers, or by consulting a third person (RP) if necessary. Then, one reviewer (AB) screened the remaining records by title and abstract. Another reviewer (FB) checked 15% of the excluded records to assess the reliability of the screening.

Next, two reviewers (AB and CB) independently screened 50 randomly selected records for full text. Any discrepancies were resolved through discussion or by consulting a third person (RP) if no agreement was reached. The remaining full-text studies were screened by one reviewer (AB). A third reviewer (FB) checked 15% of the excluded records based on the rationale for their exclusion.

The study selection process was summarised in a PRISMA flow diagram (Fig. 1).

### Data extraction

A data extraction form was developed on Google Sheets to collect general characteristics from the records, including title, nature of the record, corresponding author, and year of publication. Subsequently, for each study, we extracted information regarding the medical application, predictive model, data used to train the model, the notion of robustness addressed, and, if applicable, the use of specific metrics for assessing model robustness.

If multiple items were available for one of the characteristics listed above, each was extracted accordingly. If a study proposed and compared a machine learning model with other methods, only information on the proposed method was extracted. However, if a study evaluated the robustness of various models, details for each method were recorded.

Data extraction was initially performed by two reviewers (AB & CB) on 50 randomly selected records. Then, one reviewer (AB) completed the data extraction for all records. Subsequently, two additional reviewers (FB & OK) independently verified the extracted data for 15% of randomly selected articles each.

### Data synthesis and analysis

The extracted information was categorised as follows: each medical application was linked to a specific medical specialty with the assistance of a medical doctor; each predictive model was categorised into a broader class of machine learning methods; each data set was mapped to a type of data. One reviewer (AB) then derived general concepts to categorise the different notions of robustness extracted in each record. The concepts were chosen to encompass the different stages of a machine learning model development. The concepts were discussed and refined by discussing with another reviewer (CB), expert in machine learning.

## Supplementary information

Supplementary material

## Data Availability

Data supporting this study are available within the main article and supporting materials.

## Code availability

The screening process was conducted using Covidence systematic review software, Veritas Health Innovation, Melbourne, Australia. Available at www.covidence.org.
