# Supplementary material for: A scoping review of robustness concepts for machine learning in healthcare
Source: NPJ Digit Med. 2025 Jan 17;8:38. doi: 10.1038/s41746-024-01420-1 (PMC11742061; doi:10.1038/s41746-024-01420-1)
Supplement: Supplementary file 1 — Supplementary material [file 41746_2024_1420_MOESM1_ESM.pdf]

Supplementary Material

A scoping review of robustness concepts for machine learning in healthcare

Table of Contents

Supplementary Results....................2
Supplementary Table 1. Included studies....................2
Supplementary Table 2. List of data, model type, and medical applications classified as "Other". 22
Supplementary Table 3. Different sources of variations encountered in healthcare.................... 22
Supplementary Table 4. List of methods used to evaluate the robustness of machine learning models.................... 24
Supplementary Table 5. Concepts of robustness stratified by medical specialty....................26
Supplementary Figure 1. Alluvial diagram of data types and predictive model types.................... 27
Supplementary Table 6. Preferred Reporting Items for Systematic reviews and Meta-Analyses extension for Scoping Reviews (PRISMA-ScR) Checklist.................... 28
Supplementary Table 7. Search strategy on PubMed....................30
Supplementary Table 8. Search strategy on IEEE Xplore....................30
Supplementary Table 9. Search strategy on Web of Science.................... 30
Supplementary References....................31

Supplementary Results

Supplementary Table 1. Included studies.

Authors	Title	Medical specialty	Model type	Data modality	Concept
Karimi D, et al. (2020)	Deep learning with noisy labels: exploring techniques and remedies in medical image analysis	Urology	Deep learning	Image	Label noise
Banville H, et al. (2022)	Robust learning from corrupted EEG with dynamic spatial filtering	Neurology	Deep learning	Physiological signal	Input perturbations and alterations
Thakoor KA, et al. (2021)	Robust and Interpretable Convolutional Neural Networks to Detect Glaucoma in Optical Coherence Tomography Images	Opthalmology	Deep learning	Image	External data and domain shift
Hussein R, et al. (2018)	Optimized deep neural network architecture for robust detection of epileptic seizures using EEG signals	Neurology	Deep learning	Physiological signal	Input perturbations and alterations
Ghaffari Laleh N, et al. (2022)	Adversarial attacks and adversarial robustness in computational pathology	Urology, Gastroenterology	Deep learning	Image	Adversarial attacks
Aatresh AA, et al. (2021)	LiverNet: efficient and robust deep learning model for automatic diagnosis of sub-types of liver hepatocellular carcinoma cancer from H&E stained liver histopathology images	Gastroenterology	Deep learning	Image	External data and domain shift
Rodriguez D, et al. (2022)	On the role of deep learning model complexity in adversarial robustness for medical images	Pulmonology; Dermatology; Opthalmology	Deep learning	Image, Image, Image	Adversarial attacks
Valliani AA, et al. (2022)	Robust Prediction of Non-home Discharge After Thoracolumbar Spine Surgery With Ensemble Machine Learning and Validation on a Nationwide Cohort	Neurology	Non-deep machine learning	Clinical data	External data and domain shift
Çallı E, et al. (2021)	Deep learning with robustness to missing data: A novel approach to the detection of COVID-19	Pulmonology	Deep learning	Multimodal	Missing data
Liu W, et al. (2022)	Is the aspect ratio of cells important in deep learning? A robust comparison of deep learning methods for multi-scale cytopathology cell image classification: from convolutional neural networks to visual transformers.	ORL	Deep learning	Image	Input perturbations and alterations
Gao Y, et al. (2021)	Improving robustness of a deep learning-based lung-nodule classification model of CT images with respect to image noise	Pulmonology	Deep learning	Image	Input perturbations and alterations
Cheng S, et al. (2021)	Robust whole slide image analysis for cervical cancer screening using deep learning	ORL	Deep learning	Image	External data and domain shift
Könik A, et al. (2021)	Robustness and performance of radiomic features in diagnosing cystic renal masses	Urology	Non-deep machine learning	Image derived features	Feature extraction and selection
Liu X, et al. (2021)	An Approach for Deep Learning in ECG Classification Tasks in the Presence of Noisy Labels	Cardiology	Deep learning	Physiological signal	Label noise
Kurian NC, et al. (2021)	Robust Classification of Histology Images Exploiting Adversarial Auto Encoders	Gynaecology	Deep learning	Image	Label noise

Hsu TC, et al. (2020)	Generative Adversarial Networks for Robust Breast Cancer Prognosis Prediction with Limited Data Size	Gynaecology	Deep learning	Multimodal	Model specification and learning
Ho WH, et al. (2021)	Robust optimization of convolutional neural networks with a uniform experiment design method: a case of phonocardiogram testing in patients with heart diseases	Cardiology	Deep learning	Physiological signal	Model specification and learning
Ruano J, et al. (2022)	Robust Descriptor of Pancreatic Tissue for Automatic Detection of Pancreatic Cancer in Endoscopic Ultrasonography	Gastroenterology	Non-deep machine learning	Image derived features	Input perturbations and alterations
Zhang Y, et al. (2020)	Robustifying genomic classifiers to batch effects via ensemble learning	Infectious diseases	Non-deep machine learning	Omics	External data and domain shift
Allyn J, et al. (2020)	Adversarial attack on deep learning-based dermatoscopic image recognition systems: Risk of misdiagnosis due to undetectable image perturbations.	Dermatology	Deep learning	Image	Adversarial attacks
Amador T, et al. (2022)	Early identification of ICU patients at risk of complications: Regularization based on robustness and stability of explanations	Intensive care medicine	Non-deep machine learning	Clinical data	Model specification and learning
Joel MZ, et al. (2022)	Using Adversarial Images to Assess the Robustness of Deep Learning Models Trained on Diagnostic Images in Oncology	Pulmonology; Gynaecology; Neuro-oncology	Deep learning	Image, Image, Image	Adversarial attacks
Duggento A, et al. (2021)	A novel multi-branch architecture for state of the art robust detection of pathological phonocardiograms	Cardiology	Deep learning	Physiological signal	External data and domain shift
Cao Z, et al. (2019)	Breast tumor classification through learning from noisy labeled ultrasound images	Gynaecology	Deep learning	Image	Label noise
He L, et al. (2014)	Identifying the Gene Signatures from Gene-Pathway Bipartite Network Guarantees the Robust Model Performance on Predicting the Cancer Prognosis	Gynaecology; Hematology; Neuro-oncology	Non-deep machine learning	Omics	Model specification and learning
Kusk MW, et al. (2022)	The effect of Gaussian noise on pneumonia detection on chest radiographs, using convolutional neural networks	Pulmonology	Deep learning	Image	Input perturbations and alterations
Talmon JL, et al. (1992)	The effect of noise and biases on the performance of machine learning algorithms	Endocrinology	Non-deep machine learning	Clinical data	Label noise; Imbalanced data; Input perturbations and alterations; External data and domain shift
Gehlot S, et al. (2021)	A CNN-based unified framework utilizing projection loss in unison with label noise handling for multiple Myeloma cancer diagnosis	Hematology; Other; Pulmonology	Deep learning	Image, Image, Image	Label noise
Shi X, et al. (2019)	Graph temporal ensembling based semi-supervised convolutional neural network with noisy labels for histopathology image analysis	Pulmonology; Gynaecology	Deep learning	Image	Label noise
Kakileti ST, et al. (2020)	Robust Estimation of Breast Cancer Incidence Risk in Presence of Incomplete or Inaccurate Information	Gynaecology	Linear regression model; Non-deep machine	Clinical data	Missing data; Input perturbations and alterations

			learning; Deep learning		
Potapenko I, et al. (2021)	Detection of oedema on optical coherence tomography images using deep learning model trained on noisy clinical data	Opthalmology	Deep learning	Image	Label noise
Lim AJW, et al. (2023)	Robust SNP-based prediction of rheumatoid arthritis through machine-learning-optimized polygenic risk score	Other	Linear regression model; Non-deep machine learning; Other	Omics	Feature extraction and selection
Peng Y (2005)	A novel ensemble machine learning for robust microarray data classification	Gynaecology; Gastroenterology; Hematology; Urology	Non-deep machine learning	Omics	Model specification and learning
Mamalakis M, et al. (2021)	DenResCov-19: A deep transfer learning network for robust automatic classification of COVID-19, pneumonia, and tuberculosis from X-rays	Pulmonology	Deep learning	Image	External data and domain shift
Ren LR, et al. (2020)	L2,1-Extreme Learning Machine: An Efficient Robust Classifier for Tumor Classification	Other; Dermatology; Hematology; Gastroenterology	Deep learning	Omics	Input perturbations and alterations
Adeli E, et al. (2018)	Semi-Supervised Discriminative Classification Robust to Sample-Outliers and Feature-Noises	Neurology	Other	Image derived features, Image derived features	Input perturbations and alterations
Zhao W, et al. (2020)	A Novel Deep Neural Network for Robust Detection of Seizures Using EEG Signals	Neurology	Deep learning	Physiological signal	External data and domain shift
Yassi M, et al. (2014)	Robust and stable feature selection by integrating ranking methods and wrapper technique in genetic data classification	Urology; Other; Gastroenterology; Hematology; Neuro-oncology	Non-deep machine learning	Omics	Feature extraction and selection
Wenzel M, et al. (2019)	Automatic classification of dopamine transporter SPECT: deep convolutional neural networks can be trained to be robust with respect to variable image characteristics	Neurology	Deep learning	Image	External data and domain shift
Dyrba M, et al. (2013)	Robust Automated Detection of Microstructural White Matter Degeneration in Alzheimer's Disease Using Machine Learning Classification of Multicenter DTI Data	Neurology	Other; Non-deep machine learning	Image derived features	External data and domain shift
Qi Y, et al. (2014)	Robust Deep Network with Maximum Correntropy Criterion for Seizure Detection	Neurology	Deep learning	Physiological signal	Input perturbations and alterations

Hinrichs C, et al. (2009)	MKL for robust Multi-modality AD Classification	Neurology	Non-deep machine learning	Multimodal	Label noise
Chong DY, et al. (2015)	Robustness-driven feature selection in classification of fibrotic interstitial lung disease patterns in computed tomography using 3D texture features	Pulmonology	Non-deep machine learning	Image derived features	Feature extraction and selection
Suter Y, et al. (2020)	Radiomics for glioblastoma survival analysis in pre-operative MRI: exploring feature robustness, class boundaries, and machine learning techniques	Neuro-oncology	Non-deep machine learning; Deep learning; Linear regression model; Other	Image derived features	Feature extraction and selection
Rozycki M, et al. (2018)	Multisite Machine Learning Analysis Provides a Robust Structural Imaging Signature of Schizophrenia Detectable Across Diverse Patient Populations and Within Individuals	Psychiatry	Non-deep machine learning	Image derived features	External data and domain shift
Chaudhary K, et al. (2017)	Deep Learning–Based Multi-Omics Integration Robustly Predicts Survival in Liver Cancer	Gastroenterology	Hybrid model	Omics	External data and domain shift
Cai L, et al. (2015)	Robust phase-based texture descriptor for classification of breast ultrasound images	Gynaecology	Non-deep machine learning	Image derived features	Feature extraction and selection
Dong S, et al. (2021)	RCoNet: Deformable Mutual Information Maximization and High-Order Uncertainty-Aware Learning for Robust COVID-19 Detection	Pulmonology	Deep learning	Image	Label noise
Adeli E, et al. (2016)	Joint feature-sample selection and robust diagnosis of Parkinson's disease from MRI data	Neurology; Neurology	Other	Image derived features	Input perturbations and alterations
Moshavash Z, et al. (2018)	An Automatic and Robust Decision Support System for Accurate Acute Leukemia Diagnosis from Blood Microscopic Images	Hematology	Other; Non-deep machine learning	Image derived features	Input perturbations and alterations
Sugimoto M, et al. (2013)	Comparison of robustness against missing values of alternative decision tree and multiple logistic regression for predicting clinical data in primary breast cancer	Gynaecology	Non-deep machine learning; Linear regression model	Clinical data	Missing data
Mayer RS, et al. (2022)	How to learn with intentional mistakes: NoisyEnsembles to overcome poor tissue quality for deep learning in computational pathology	Gynaecology	Deep learning	Image	Input perturbations and alterations
Maron RC, et al. (2021)	Robustness of convolutional neural networks in recognition of pigmented skin lesions	Dermatology	Deep learning	Image	Input perturbations and alterations
Mi H, et al. (2015)	Robust feature selection to predict tumor treatment outcome	Pulmonology; Gastroenterology	Non-deep machine learning	Multimodal	Feature extraction and selection

Khan MA, et al. (2020)	Multimodal Brain Tumor Classification Using Deep Learning and Robust Feature Selection: A Machine Learning Application for Radiologists	Neuro-oncology	Deep learning	Image derived features	Feature extraction and selection
Venton J, et al. (2021)	Robustness of convolutional neural networks to physiological electrocardiogram noise	Cardiology	Deep learning	Physiological signal	Input perturbations and alterations
Hsu TC, et al. (2023)	Learning from small medical data-robust semi-supervised cancer prognosis classifier with Bayesian variational autoencoder.	Gynaecology; Pulmonology	Deep learning	Multimodal	External data and domain shift; Model specification and learning
Ren Q, et al. (2022)	Assessing the robustness of radiomics/deep learning approach in the identification of efficacy of anti–PD-1 treatment in advanced or metastatic non-small cell lung carcinoma patients	Pulmonology	Linear regression model; Non-deep machine learning; Deep learning; Other; Hybrid model	Image derived features	Feature extraction and selection
Alessandrini M, et al. (2022)	EEG-Based Alzheimer's Disease Recognition Using Robust-PCA and LSTM Recurrent Neural Network	Neurology	Deep learning	Physiological signal	Input perturbations and alterations
Chuah J, et al. (2022)	Framework for Testing Robustness of Machine Learning-Based Classifiers	Neurology	Other; Linear regression model; Non-deep machine learning; Deep learning	Omics	Feature extraction and selection; Input perturbations and alterations; Model specification and learning
Trivizakis E, et al. (2020)	Advancing COVID-19 differentiation with a robust preprocessing and integration of multi-institutional open-repository computer tomography datasets for deep learning analysis	Pulmonology	Deep learning	Image	Input perturbations and alterations
Fatema K, et al. (2022)	A Robust Framework Combining Image Processing and Deep Learning Hybrid Model to Classify Cardiovascular Diseases Using a Limited Number of Paper-Based Complex ECG Images	Cardiology	Deep learning	Physiological signal	Model specification and learning
Jang R, et al. (2020)	Assessment of the Robustness of Convolutional Neural Networks in Labeling Noise by Using Chest X-Ray Images From Multiple Centers	Pulmonology	Deep learning	Image	Label noise
Hammad M, et al. (2022)	Efficient multimodal deep-learning-based COVID-19 diagnostic system for noisy and corrupted images	Pulmonology	Deep learning	Image	Input perturbations and alterations
Hashemzehi R, et al. (2021)	Y-net: a reducing gaussian noise convolutional neural network for MRI brain tumor classification with NADE concatenation	Neuro-oncology	Deep learning	Image	Input perturbations and alterations
Massafra R, et al. (2022)	Robustness Evaluation of a Deep Learning Model on Sagittal and Axial Breast DCE-MRIs to Predict Pathological Complete Response to Neoadjuvant Chemotherapy	Gynaecology	Hybrid model	Multimodal	External data and domain shift
Hekler A, et al. (2020)	Effects of Label Noise on Deep Learning-Based Skin Cancer Classification	Dermatology	Deep learning	Image	Label noise

Ma L, et al. (2022)	A regularization method to improve adversarial robustness of neural networks for ECG signal classification	Cardiology	Deep learning	Physiological signal	Adversarial attacks
Itoh H, et al. (2020)	Robust endocytoscopic image classification based on higher-order symmetric tensor analysis and multi-scale topological statistics	Gastroenterology	Non-deep machine learning	Image derived features	External data and domain shift
Liu J, et al. (2023)	AI-Driven Robust Kidney and Renal Mass Segmentation and Classification on 3D CT Images	Urology	Deep learning	Image	External data and domain shift
Liu J, et al. (2021)	Co-Correcting: Noise-tolerant Medical Image Classification via mutual Label Correction	Dermatology; Other	Deep learning	Image, Image	Label noise
Montaha S, et al. (2022)	MNet-10: A robust shallow convolutional neural network model performing ablation study on medical images assessing the effectiveness of applying optimal data augmentation technique.	Gynaecology; Gynaecology; Pulmonology; Dermatology; Neuro-oncology; Gynaecology; Pulmonology; ORL	Deep learning	Image, Image, Image, Image, Image, Image, Image, Image	External data and domain shift; Model specification and learning
Mi Z, et al. (2010)	Module-based prediction approach for robust inter-study predictions in microarray data	Urology; Pulmonology	Other; Non-deep machine learning	Omics	Input perturbations and alterations; External data and domain shift
Monday HN, et al. (2022)	COVID-19 Diagnosis from Chest X-ray Images Using a Robust Multi-Resolution Analysis Siamese Neural Network with Super-Resolution Convolutional Neural Network	Pulmonology	Deep learning	Image	Input perturbations and alterations
Ghosh-Dastidar S, et al. (2008)	Principal Component Analysis-Enhanced Cosine Radial Basis Function Neural Network for Robust Epilepsy and Seizure Detection	Neurology	Deep learning	Physiological signal	Model specification and learning
Wei Z, et al. (2022)	Deep Learning-Based Multi-Omics Integration Robustly Predicts Relapse in Prostate Cancer	Urology	Hybrid model	Omics	External data and domain shift
Foote A, et al. (2022)	REET: robustness evaluation and enhancement toolbox for computational pathology	Other	Deep learning	Image	Input perturbations and alterations; Adversarial attacks
Almalki YE, et al. (2022)	Robust Gaussian and Nonlinear Hybrid Invariant Clustered Features Aided Approach for Speeded Brain Tumor Diagnosis	Neuro-oncology	Non-deep machine learning	Image derived features	Feature extraction and selection
Pierce SG, et al. (2006)	Evaluation of Neural Network Robust Reliability Using Information-Gap Theory	Gynaecology	Deep learning	Image derived features	Model specification and learning
Ubaldi L, et al. (2023)	Deriving quantitative information from multiparametric MRI via Radiomics: Evaluation of the robustness and predictive value of radiomic features in the discrimination of low-grade versus high-grade gliomas with machine learning	Neuro-oncology	Non-deep machine learning	Image derived features	Feature extraction and selection
Liu X, et al. (2021)	VidAF: A Motion-Robust Model for Atrial Fibrillation Screening From Facial Videos	Cardiology	Deep learning	Physiological signal	Input perturbations and alterations

Donnelly-Kehoe PA, et al. (2019)	Robust automated computational approach for classifying frontotemporal neurodegeneration: Multimodal/multicenter neuroimaging	Neurology	Non-deep machine learning	Image derived features	External data and domain shift
Weninger L, et al. (2019)	Robustness of Radiomics for Survival Prediction of Brain Tumor Patients Depending on Resection Status	Neuro-oncology	Linear regression model; Non-deep machine learning	Image derived features	Feature extraction and selection
Katsch F, et al. (2022)	Comparison of Convolutional Neural Network Architectures for Robustness Against Common Artefacts in Dermatoscopic Images	Dermatology	Deep learning	Image	Input perturbations and alterations
Duo M, et al. (2022)	Integrative bioinformatics analysis to explore a robust diagnostic signature and landscape of immune cell infiltration in sarcoidosis	Other	Linear regression model	Omics	External data and domain shift
O'Connell GC, et al. (2017)	Stroke-associated pattern of gene expression previously identified by machine-learning is diagnostically robust in an independent patient population	Neurology	Non-deep machine learning	Omics	External data and domain shift
Guan Y, et al. (2021)	Assessment of the timeliness and robustness for predicting adult sepsis	Infectious diseases	Non-deep machine learning	Clinical data	External data and domain shift
Ho JC, et al. (2017)	Learning from Different Perspectives: Robust Cardiac Arrest Prediction via Temporal Transfer Learning	Intensive care medicine	Linear regression model	Clinical data	Model specification and learning
Sraitih M, et al. (2022)	A Robustness Evaluation of Machine Learning Algorithms for ECG Myocardial Infarction Detection	Cardiology	Non-deep machine learning	Physiological signal	Input perturbations and alterations
Whitney HM, et al. (2021)	Multi-Stage Harmonization for Robust AI across Breast MR Databases	Gynaecology	Other	Image derived features	Feature extraction and selection
Bhanot G, et al. (2005)	A robust meta-classification strategy for cancer detection from MS data	Urology	Hybrid model	Omics	Feature extraction and selection
Engemann DA, et al. (2018)	Robust EEG-based cross-site and cross-protocol classification of states of consciousness	Neurology	Non-deep machine learning	Physiological signal	External data and domain shift; Label noise; Input perturbations and alterations
Neeb H, et al. (2018)	Multivariate prediction of multiple sclerosis using robust quantitative MR-based image metrics	Neurology	Non-deep machine learning	Image derived features	Input perturbations and alterations
Yang Y, et al. (2021)	Robust Collaborative Learning of Patch-level and Image-level Annotations for Diabetic Retinopathy Grading from Fundus Image	Opthalmology	Deep learning	Image	External data and domain shift
Poirot MG, et al. (2022)	Robustness of radiomics to variations in segmentation methods in multimodal brain MRI	Neurology	Deep learning	Image derived features	Feature extraction and selection

Xu M, et al. (2021)	Towards evaluating the robustness of deep diagnostic models by adversarial attack	Dermatology; Opthalmology; Pulmonology	Deep learning	Image, Image, Image	Adversarial attacks; Input perturbations and alterations
Sudjai N, et al. (2023)	Robustness of Radiomic Features: Two-Dimensional versus Three-Dimensional MRI-Based Feature Reproducibility in Lipomatous Soft-Tissue Tumors	Other	Linear regression model; Non-deep machine learning	Image derived features	Feature extraction and selection
Kollen B, et al. (2005)	Longitudinal robustness of variables predicting independent gait following severe middle cerebral artery stroke: a prospective cohort study	Neurology	Linear regression model	Clinical data	External data and domain shift
Gallego Vázquez C, et al. (2022)	Label noise and self-learning label correction in cardiac abnormalities classification	Cardiology	Deep learning	Physiologica l signal	Label noise
Yang M, et al. (2022)	Performance improvement in multi-label thoracic abnormality classification of chest X-rays with noisy labels	Pulmonology	Deep learning	Image	Label noise
Huo Z, et al. (2000)	Computerized Classification of Benign and Malignant Masses on Digitized Mammograms: A Study of Robustness	Gynaecology	Deep learning	Image derived features	External data and domain shift
Adnan N, et al. (2022)	A Robust Personalized Classification Method for Breast Cancer Metastasis Prediction	Gynaecology	Linear regression model	Omics	Feature extraction and selection
Kyung S, et al. (2022)	Improved performance and robustness of multi-task representation learning with consistency loss between pretexts for intracranial hemorrhage identification in head CT	Neurology	Deep learning	Image	External data and domain shift
Ehsani R, et al. (2020)	Robust Distance Measures for kNN Classification of Cancer Data	Neuro-oncology; Gynaecology	Non-deep machine learning	Image, Image derived features	Model specification and learning
Schiavi S, et al. (2022)	Classification of multiple sclerosis patients based on structural disconnection: A robust feature selection approach	Neurology	Non-deep machine learning	Image derived features	Feature extraction and selection
Castro-Luna GM, et al. (2019)	Robust keratoconus detection with Bayesian network classifier for Placido-based corneal indices	Opthalmology	Other	Other	Input perturbations and alterations
Cao XH, et al. (2016)	A robust data scaling algorithm to improve classification accuracies in biomedical data	Gastroenterology; Urology; Pulmonology; Gynaecology; Hematology; Gynaecology;	Linear regression model; Non-deep machine learning	Omics, Omics, Omics, Omics, Omics, Other,	Input perturbations and alterations

		Neurology; Other; Neurology; Gynaecology; Gastroenterology; Endocrinology		Other, Omics, Other, Image derived features, Other, Clinical data	
Olsen M, et al. (2019)	Robust, ECG-based detection of Sleep-disordered breathing in large population-based cohorts	Neurology	Deep learning	Physiological signal	External data and domain shift
Pratap T, et al. (2020)	Efficient network selection for computer-aided cataract diagnosis under noisy environment	Opthalmology	Hybrid model	Image	Input perturbations and alterations
Chen W, et al. (2010)	Computerized assessment of breast lesion malignancy using DCE-MRI: robustness study on two independent clinical datasets from two manufacturers	Gynaecology	Deep learning	Image derived features	External data and domain shift
Anghel A, et al. (2019)	A High-Performance System for Robust Stain Normalization of Whole-Slide Images in Histopathology	Urology	Deep learning	Image	Input perturbations and alterations
Guo LL, et al. (2022)	Evaluation of domain generalization and adaptation on improving model robustness to temporal dataset shift in clinical medicine.	Intensive care medicine	Deep learning	Clinical data	External data and domain shift
Ying X, et al. (2023)	COVID-19 chest X-ray image classification in the presence of noisy labels	Pulmonology	Deep learning	Image	Label noise
Li H, et al. (2012)	Computerized Analysis of Mammographic Parenchymal Patterns on a Large Clinical Dataset of Full-Field Digital Mammograms: Robustness Study with Two High-Risk Datasets	Gynaecology	Deep learning	Image derived features	External data and domain shift
Iori, M, et al. (2022)	Mortality Prediction of COVID-19 Patients Using Radiomic and Neural Network Features Extracted from a Wide Chest X-ray Sample Size: A Robust Approach for Different Medical Imbalanced Scenarios	Pulmonology	Linear regression model; Other; Non-deep machine learning	Image derived features	Imbalanced data
Khosravi, M, et al. (2022)	A Robust Machine learning based method to classify normal and abnormal CT scan images of mastoid air cells	ORL	Deep learning	Image	Model specification and learning
Pratap, T, et al. (2021)	Deep neural network based robust computer-aided cataract diagnosis system using fundus retinal images	Opthalmology	Hybrid model	Image derived features	Input perturbations and alterations
Hallaji, E, et al. (2021)	Adversarial Learning on Incomplete and Imbalanced Medical Data for Robust Survival Prediction of Liver Transplant Patients	Gastroenterology	Deep learning	Clinical data	Imbalanced data; Missing data
Qi, Y, et al. (2022)	Learning Robust Features From Nonstationary Brain Signals by Multiscale Domain Adaptation Networks for Seizure Prediction	Neurology	Deep learning	Physiological signal	External data and domain shift
Back, S, et al. (2021)	Robust Skin Disease Classification by Distilling Deep Neural Network Ensemble for the Mobile Diagnosis of Herpes Zoster	Dermatology	Deep learning	Image	Input perturbations and alterations

Chen, X, et al. (2022)	AutoMO-Mixer: An Automated Multi-objective Mixer Model for Balanced, Safe and Robust Prediction in Medicine	Opthalmology	Deep learning	Image	Adversarial attacks
Alzubaidi, L, et al. (2021)	Robust application of new deep learning tools: an experimental study in medical imaging	Gynaecology; Other; Other	Deep learning	Image, Image, Image	External data and domain shift
Hogue, MA, et al. (2021)	Investigating the Robustness of Deep Neural Network Based COVID-19 Detection Models Against Universal Adversarial Attacks	Pulmonology	Deep learning	Image	Adversarial attacks
Organisciak, D, et al. (2022)	RobIn: A robust interpretable deep network for schizophrenia diagnosis	Psychiatry	Deep learning	Clinical data	Input perturbations and alterations; Missing data
Hassan, T, et al. (2022)	Ultrasound image augmentation by tumor margin appending for robust deep learning based breast lesion classification	Gynaecology	Deep learning	Image	Input perturbations and alterations
Yun, S, et al. (2021)	Robust Deep Multi-task Learning Framework for Cancer Survival Analysis	Other	Deep learning	Multimodal	Imbalanced data
Almalik, F, et al. (2022)	Self-Ensembling Vision Transformer (SEViT) for Robust Medical Image Classification	Infectious diseases; Opthalmology	Deep learning	Image, Image	Adversarial attacks
Hajiabadi, H, et al. (2020)	Combination of loss functions for robust breast cancer prediction	Gynaecology	Deep learning	Image derived features	Label noise
Clancy, K, et al. (2019)	Deep learning for identifying breast cancer malignancy and false recalls: A robustness study on training strategy	Gynaecology	Deep learning	Image	Model specification and learning
Daanouni, O, et al. (2022)	NSL-MHA-CNN: A Novel CNN Architecture for Robust Diabetic Retinopathy Prediction Against Adversarial Attacks	Opthalmology	Deep learning	Image	Adversarial attacks
Jaskari, J, et al. (2022)	Uncertainty-Aware Deep Learning Methods for Robust Diabetic Retinopathy Classification	Opthalmology	Deep learning	Image	Model specification and learning
Huq, A, et al. (2020)	Robust Deep Neural Network Model for Identification of Malaria Parasites in Cell Images	Infectious diseases	Deep learning	Image	Adversarial attacks
Anand, D, et al. (2020)	Self-Supervision vs. Transfer Learning: Robust Biomedical Image Analysis Against Adversarial Attacks	Pulmonology	Deep learning	Image	Adversarial attacks
Maron, RC, et al. (2021)	A benchmark for neural network robustness in skin cancer classification	Dermatology	Deep learning	Image	Input perturbations and alterations
Mishra, S, et al. (2021)	Robustness of Deep Learning Models in Dermatological Evaluation: A Critical Assessment	Dermatology	Deep learning	Image	Input perturbations and alterations; External data and domain shift
Park, K, et al. (2013)	Robust predictive model for evaluating breast cancer survivability	Gynaecology	Non-deep machine learning; Deep learning; Other	Clinical data	Model specification and learning
Yang, ZB, et al. (2020)	Accurate and adversarially robust classification of medical images and ECG time-series with gradient-free trained sign activation neural networks	Pulmonology; Cardiology; Gastroenterology	Deep learning	Image, Physiological signal, Image	Adversarial attacks

Thomas, AH, et al. (2020)	Noise-Resilient and Interpretable Epileptic Seizure Detection	Neurology	Deep learning	Physiological signal	Input perturbations and alterations
Akbarimajd, A, et al. (2022)	Learning-to-augment incorporated noise-robust deep CNN for detection of COVID-19 in noisy X-ray images	Pulmonology	Deep learning	Image	Input perturbations and alterations
Chen, L, et al. (2020)	Graph Learning Approaches for Graph with Noise: Application to Disease Prediction in Population Graph	Neurology	Deep learning	Image derived features	Label noise
Xue, C, et al. (2022)	Robust Medical Image Classification from Noisy Labeled Data with Global and Local Representation Guided Co-training	Dermatology; Other; Pulmonology; Urology	Deep learning	Image, Image, Image, Image	Label noise
Wang, K, et al. (2019)	How Robust is Your Automatic Diagnosis Model?	Intensive care medicine	Deep learning	Other	Adversarial attacks
Zhang, YL, et al. (2022)	Benchmarking the Robustness of Deep Neural Networks to Common Corruptions in Digital Pathology	Other; Gynaecology	Deep learning	Image	Input perturbations and alterations
Chatterjee, A, et al. (2019)	Creating Robust Predictive Radiomic Models for Data From Independent Institutions Using Normalization	Gynaecology	Linear regression model	Image derived features	External data and domain shift
Lu, Y, et al. (2020)	Robust Speech and Natural Language Processing Models for Depression Screening	Psychiatry	Deep learning	Other	External data and domain shift
Ochoa, A, et al. (2019)	Noise-tolerant Modular Neural Network System for Classifying ECG Signal	Cardiology	Deep learning	Physiological signal	Input perturbations and alterations
Subbaswamy, A, et al. (2021)	Evaluating Model Robustness and Stability to Dataset Shift	Infectious diseases	Non-deep machine learning	Clinical data	External data and domain shift
Wang, Z (2018)	Robust boosting with truncated loss functions	Gynaecology	Non-deep machine learning	Omics	Label noise
Ren, HX, et al. (2021)	RAPT: Pre-training of Time-Aware Transformer for Learning Robust Healthcare Representation	Gynaecology	Deep learning	Clinical data	External data and domain shift
Xue, C, et al. (2019)	Robust Learning at Noisy Labeled Medical Images: Applied to Skin Lesion Classification	Dermatology	Deep learning	Image	Label noise
O'Brien, M, et al. (2022)	Evaluating Neural Network Robustness for Melanoma Classification using Mutual Information	Dermatology	Deep learning	Image	External data and domain shift
Nurmaini, S, et al. (2020)	Robust detection of atrial fibrillation from short-term electrocardiogram using convolutional neural networks	Cardiology	Deep learning	Physiological signal	External data and domain shift
Arcaini, P, et al. (2020)	Dealing with Robustness of Convolutional Neural Networks for Image Classification	Gynaecology	Deep learning	Image	Input perturbations and alterations
Petersen, E, et al. (2022)	Feature robustness and sex differences in medical imaging: a case study in MRI-based Alzheimer's disease detection	Neurology	Deep learning; Linear regression model	Image derived features, Image	External data and domain shift

Kamran, SA, et al. (2020)	Improving Robustness Using Joint Attention Network for Detecting Retinal Degeneration From Optical Coherence Tomography Images	Opthalmology	Deep learning	Image	External data and domain shift
Xie, L, et al. (2020)	Towards implementation of AI in New Zealand national diabetic screening program: Cloudbased, robust, and bespoke	Opthalmology	Deep learning	Image	Input perturbations and alterations
Zhu, MJ, et al. (2022)	Robust co-teaching learning with consistency-based noisy label correction for medical image classification	Dermatology; Endocrinology	Deep learning	Image, Image	Label noise
Pang, CY, et al. (2022)	Improving model robustness via enhanced feature representation and sample distribution based on cascaded classifiers for computer-aided diagnosis of brain disease	Neurology; Neurology	Deep learning	Image derived features	Model specification and learning
Abbas, MR, et al. (2019)	Accuracy Rejection Normalized-Cost Curves (ARNCCs): A Novel 3-Dimensional Framework for Robust Classification	Gynaecology; Gynaecology	Other; Linear regression model	Image derived features, Clinical data	Model specification and learning
Malafaia, M, et al. (2022)	Robustness Analysis of Deep Learning-Based Lung Cancer Classification Using Explainable Methods	Pulmonology	Deep learning	Image	Model specification and learning
Li, X, et al. (2020)	Robust Detection of Adversarial Attacks on Medical Images	Pulmonology	Deep learning	Image	Adversarial attacks
Beyrami, SMG, et al. (2020)	A robust, cost-effective and non-invasive computer-aided method for diagnosis three types of neurodegenerative diseases with gait signal analysis	Neurology	Other	Other	External data and domain shift
Shanthini, A, et al. (2019)	A taxonomy on impact of label noise and feature noise using machine learning techniques	Gynaecology	Non-deep machine learning	Image derived features	Input perturbations and alterations; Label noise
Wahi-Anwar, MW, et al. (2021)	A Novel Physics-based Data Augmentation Approach for Improved Robust Deep Learning in Medical Imaging: Lung Nodule CAD False Positive Reduction in Low-Dose CT Environments	Pulmonology	Deep learning	Image	External data and domain shift
Waseem, MH, et al. (2019)	On the Feature Selection Methods and Reject Option Classifiers for Robust Cancer Prediction	Hematology; Gastroenterology; Gynaecology	Other; Linear regression model; Non-deep machine learning	Omics	Feature extraction and selection; Model specification and learning
Kurian, NC, et al. (2022)	Improved Histology Image Classification under Label Noise Via Feature Aggregating Memory Banks	Gynaecology	Deep learning	Image, Image	Label noise
Booth, BM, et al. (2022)	Toward Robust Stress Prediction in the Age of Wearables: Modeling Perceived Stress in a Longitudinal Study with Information Workers	Psychiatry	Linear regression model; Non-deep machine learning; Deep learning	Other	Label noise; Imbalanced data; Missing data

Hussein, R, et al. (2018)	Robust detection of epileptic seizures based on L1-penalized robust regression of EEG signals	Neurology	Non-deep machine learning	Physiological signal	Input perturbations and alterations
Xue, FF, et al. (2019)	Improving Robustness of Medical Image Diagnosis with Denoising Convolutional Neural Networks	Dermatology; Pulmonology	Deep learning	Image, Image	Adversarial attacks
Lee, H, et al. (2022)	Noisy Label Classification using Label Noise Selection with Test-Time Augmentation Cross-Entropy and NoiseMix Learning	Dermatology	Deep learning	Image	Label noise
Das, SSS, et al. (2022)	BayesBeat: Reliable Atrial Fibrillation Detection from Noisy Photoplethysmography Data	Cardiology	Deep learning	Physiological signal	Input perturbations and alterations
Samarasinghe, S (2016)	Order in the Black Box: Consistency and Robustness of Hidden Neuron Activation of Feed Forward Neural Networks and Its Use in Efficient Optimization of Network Structure	Gynaecology	Deep learning	Clinical data	Model specification and learning
Mata, D, et al. (2022)	Increased Robustness in Chest X-Ray Classification Through Clinical Report-Driven Regularization	Pulmonology	Deep learning	Multimodal	Model specification and learning
Gouabou, ACF, et al. (2022)	End-to-End Decoupled Training: A Robust Deep Learning Method for Long-Tailed Classification of Dermoscopic Images for Skin Lesion Classification	Dermatology	Deep learning	Image	Imbalanced data
Soukup, M, et al. (2005)	Robust classification modeling on microarray data using misclassification penalized posterior	Hematology; Gastroenterology	Other; Linear regression model; Non-deep machine learning	Omics	Model specification and learning
dos Santos, FP, et al. (2018)	Robust feature spaces from pre-trained deep network layers for skin lesion classification	Dermatology	Hybrid model	Image	Feature extraction and selection
Conroy, B, et al. (2015)	A dynamic ensemble approach to robust classification in the presence of missing data	Intensive care medicine	Non-deep machine learning	Clinical data	Missing data
Arcaini, P, et al. (2021)	ROBY: a Tool for Robustness Analysis of Neural Network Classifiers	Gynaecology	Deep learning	Image	Input perturbations and alterations
Kamran, SA, et al. (2022)	Feature Representation Learning for Robust Retinal Disease Detection from Optical Coherence Tomography Images	Opthalmology	Deep learning	Image	External data and domain shift
Feng, XX, et al. (2022)	Robust Classification Model for Diabetic Retinopathy Based on the Contrastive Learning Method with a Convolutional Neural Network	Opthalmology	Deep learning	Image	Model specification and learning
Songyang, YY, et al. (2019)	Large-scale gene expression analysis reveals robust gene signatures for prognosis prediction in lung adenocarcinoma	Pulmonology	Linear regression model	Omics	External data and domain shift
Shi, XS, et al. (2022)	Robust convolutional neural networks against adversarial attacks on medical images	Pulmonology; Opthalmology	Deep learning	Image, Image	Adversarial attacks
Khoshnevisan, F, et al. (2021)	Unifying Domain Adaptation and Domain Generalization for Robust Prediction across Minority Racial Groups	Intensive care medicine	Deep learning	Clinical data	External data and domain shift

Song, WT, et al. (2021)	A Statistical Robust Glaucoma Detection Framework Combining Retinex, CNN, and DOE Using Fundus Images	Opthalmology	Deep learning	Image	Model specification and learning
Karagoz, A, et al. (2022)	Robust whole-tumour 3D volumetric CT-based radiomics approach for predicting the WHO/ISUP grade of a ccRCC tumour	Urology	Non-deep machine learning	Image derived features	Input perturbations and alterations
Oliveira, C, et al. (2021)	Preselection of robust radiomic features does not improve outcome modelling in non-small cell lung cancer based on clinical routine FDG-PET imaging	Pulmonology	Linear regression model	Image derived features	Feature extraction and selection
Zuo, SG, et al. (2019)	A robust six-gene prognostic signature for prediction of both disease-free and overall survival in non-small cell lung cancer	Pulmonology	Linear regression model	Omics	Model specification and learning
Xi, XM, et al. (2017)	Robust texture analysis of multi-modal images using Local Structure Preserving Ranklet and multi-task learning for breast tumor diagnosis	Gynaecology	Non-deep machine learning	Image derived features	Feature extraction and selection
Pan, SY, et al. (2022)	BAW: Learning from class imbalance and noisy labels with Batch Adaptation Weighted Loss	Pulmonology	Deep learning	Image	Label noise; Imbalanced data
Lebedev, AV, et al. (2014)	Random Forest ensembles for detection and prediction of Alzheimer's disease with a good between-cohort robustness	Neurology	Non-deep machine learning	Image derived features	External data and domain shift
Swami, P, et al. (2016)	A novel robust diagnostic model to detect seizures in electroencephalography	Neurology	Deep learning	Physiological signal	Model specification and learning
Madruga, M, et al. (2020)	Multicondition training for noise-robust detection of benign vocal fold lesions from recorded speech	ORL	Non-deep machine learning	Other	Input perturbations and alterations
Wang, K, et al. (2022)	Robust Identification of Subtypes in Non-Small Cell Lung Cancer Using Radiomics	Pulmonology	Non-deep machine learning; Linear regression model	Image derived features	Feature extraction and selection
Xu, MT, et al. (2022)	MedRDF: A Robust and Retrain-Less Diagnostic Framework for Medical Pretrained Models Against Adversarial Attack	Pulmonology; Dermatology	Deep learning	Image, Image	Input perturbations and alterations; Adversarial attacks
Champion, A, et al. (2014)	Semantic interpretation of robust imaging features for Fuhrman grading of renal carcinoma	Urology	Non-deep machine learning	Image derived features	Model specification and learning
Wang, X, et al. (2022)	SurvMaximin: Robust federated approach to transporting survival risk prediction models	Other	Linear regression model	Clinical data	Model specification and learning
Ray, S, et al. (2022)	A robust COVID-19 mortality prediction calculator based on Lymphocyte count, Urea, C-Reactive Protein, Age and Sex (LUCAS) with chest X-rays	Pulmonology	Linear regression model	Clinical data	External data and domain shift
Pratap, T, et al. (2020)	Correcting Automatic Cataract Diagnosis Systems Against Noisy/Blur Environment	Opthalmology	Hybrid model	Image	Input perturbations and alterations

Vargason, T, et al. (2020)	Classification of autism spectrum disorder from blood metabolites: Robustness to the presence of co-occurring conditions	Neurology	Other	Omics	External data and domain shift
Zhao, JH, et al. (2021)	Noisy Mammogram Classification Method Based on New Weighted Fusion Framework	Gynaecology	Deep learning	Image	Input perturbations and alterations
Ramoni, M, et al. (2001)	Robust Outcome Prediction for Intensive-Care Patients	Intensive care medicine	Other	Clinical data	Missing data
Lausser, L, et al. (2020)	Constraining classifiers in molecular analysis: invariance and robustness	Hematology; Gynaecology; Urology; Other; Neurology; Gastroenterology; Pulmonology; Dermatology	Non-deep machine learning	Omics	Input perturbations and alterations
Binta, N, et al. (2022)	Hilbert-Envelope Features for Cardiac Disease Classification from Noisy Phonocardiograms	Cardiology	Hybrid model	Physiological signal	Input perturbations and alterations
Li, JT, et al. (2022)	Improving diagnosis accuracy of non-small cell lung carcinoma on noisy data by adaptive group lasso regularized multinomial regression	Pulmonology	Linear regression model	Omics	Input perturbations and alterations
Sbrollini, A, et al. (2021)	Repeated Structuring & Learning Procedure for Detection of Myocardial Ischemia: a Robustness Analysis	Cardiology	Deep learning	Physiological signal	Model specification and learning
Gruszauskas, NP, et al. (2009)	Breast US Computer-aided Diagnosis System: Robustness across Urban Populations in South Korea and the United States	Gynaecology	Deep learning	Image derived features	External data and domain shift
Fengbei, LB, et al. (2022)	NVUM: Non-Volatile Unbiased Memory for Robust Medical Image Classification	Pulmonology	Deep learning	Image	Label noise; Imbalanced data
Mitra, V, et al. (2016)	Noise and reverberation effects on depression detection from speech	Psychiatry	Non-deep machine learning; Deep learning	Other	Input perturbations and alterations
Bhattacharjee, T, et al. (2021)	Effect of Noise and Model Complexity on Detection of Amyotrophic Lateral Sclerosis and Parkinson's Disease Using Pitch and MFCC	Neurology; Neurology	Deep learning	Other	Input perturbations and alterations
Huo, JY, et al. (2021)	Development and Validation of a Robust Immune-Related Prognostic Signature for Gastric Cancer	Gastroenterology	Linear regression model	Omics	External data and domain shift
Zhang, W, et al. (2022)	A Novel and Robust Prognostic Model for Hepatocellular Carcinoma Based on Enhancer RNAs-Regulated Genes	Gastroenterology	Linear regression model	Omics	External data and domain shift
Wu, PC, et al. (2020)	Development and validation of a robust immune-related prognostic signature in early-stage lung adenocarcinoma	Pulmonology	Linear regression model	Omics	External data and domain shift
T. Peng, et al. (2020)	Noise Robust Learning with Hard Example Aware for Pathological Image classification	Gastroenterology; Gastroenterology	Deep learning	Image	Label noise

Y. Cao, et al. (2021)	An auxiliary tool for preliminary tests of skin cancer : A self-modifying meta-learning method for clean and noisy data	Dermatology	Deep learning	Image	Label noise
R. Colbaugh, et al. (2018)	Learning to Identify Rare Disease Patients from Electronic Health Records	Other	Hybrid model	Clinical data	Label noise
A. Majumdar, et al. (2017)	Robust Greedy Deep Dictionary Learning for ECG Arrhythmia Classification	Cardiology	Hybrid model	Physiological signal	Input perturbations and alterations
D. Padovano, et al. (2022)	Machine Learning Methods to Detect Obstructive Sleep Apnea under a Robust Testing Framework	Neurology	Non-deep machine learning	Physiological signal	External data and domain shift
S. Chuprov, et al. (2022)	Are ML Image Classifiers Robust to Medical Image Quality Degradation?	Pulmonology	Deep learning	Image	Input perturbations and alterations
P. J. Garcia-Laencina, et al. (2008)	A robust approach for classifying unknown data in medical diagnosis problems	Gynaecology	Non-deep machine learning	Clinical data	Missing data
I. Escrivães, et al. (2022)	ECG classification using Artificial Intelligence: Model Optimization and Robustness Assessment.	Cardiology	Deep learning	Physiological signal	Model specification and learning
J. Roh (2022)	Impact of Adversarial Training on the Robustness of Deep Neural Networks	Other	Non-deep machine learning; Deep learning	Image	Input perturbations and alterations; Adversarial attacks
Y. Yang, et al. (2022)	A robust and generalizable immune-related signature for sepsis diagnostics	Infectious diseases	Linear regression model	Omics	External data and domain shift
N. C. Kurian, et al. (2021)	Sample Specific Generalized Cross Entropy for Robust Histology Image Classification	Gynaecology	Deep learning	Image	Label noise
K. Ren, et al. (2018)	A Robust AUC Maximization Framework With Simultaneous Outlier Detection and Feature Selection for Positive-Unlabeled Classification	Infectious diseases; Neurology	Other	Clinical data, Physiological signal	Imbalanced data; Input perturbations and alterations; Label noise
K. Shekokar, et al. (2022)	Identification of Epileptic Seizures using CNN on Noisy EEG Signals	Neurology	Deep learning	Physiological signal	Input perturbations and alterations
W. A. Al-Olofi, et al. (2018)	Improved Anomaly Detection in Low-Resolution and Noisy Whole-Slide Images using Transfer Learning	Gynaecology	Non-deep machine learning	Image derived features	Input perturbations and alterations
A. Jaiswal, et al. (2022)	RoS-KD: A Robust Stochastic Knowledge Distillation Approach for Noisy Medical Imaging	Dermatology; Pulmonology	Deep learning	Image, Image	Adversarial attacks
R. Venkatesh, et al. (2006)	Robust Model Selection Using Cross Validation: A Simple Iterative Technique for Developing Robust Gene Signatures in Biomedical Genomics Applications	Gastroenterology	Other	Omics	Feature extraction and selection
S. Amin, et al. (2016)	A robust approach towards epileptic seizure detection	Neurology	Non-deep machine learning	Physiological signal	Imbalanced data

M. Osman, et al. (2022)	SkinFormer: Robust Vision Transformer for Automatic Skin Disease Identification	Dermatology	Deep learning	Image	External data and domain shift
P. Arcaini, et al. (2021)	Efficient Computation of Robustness of Convolutional Neural Networks	Gynaecology	Deep learning	Image	Input perturbations and alterations
Y. -C. Chuang, et al. (2020)	An Arbitrarily Reconfigurable Extreme Learning Machine Inference Engine for Robust ECG Anomaly Detection	Cardiology	Hybrid model	Physiological signal	Input perturbations and alterations
X. Fan, et al. (2020)	Effect of Image Noise on the Classification of Skin Lesions Using Deep Convolutional Neural Networks	Dermatology	Deep learning	Image	Input perturbations and alterations
Y. -L. Huang, et al. (2020)	Conditional Domain Adversarial Transfer for Robust Cross-Site ADHD Classification Using Functional MRI	Neurology	Deep learning	Image derived features	External data and domain shift
B. -F. Wu, et al. (2022)	Motion-Robust Atrial Fibrillation Detection Based on Remote-Photoplethysmography	Cardiology	Deep learning	Physiological signal	Input perturbations and alterations
R. Ghongade, et al. (2008)	A Robust and Reliable ECG Pattern Classification using QRS Morphological Features and ANN	Cardiology	Deep learning	Physiological signal	Input perturbations and alterations
Y. Park, et al. (2019)	Tackling Overfitting in Boosting for Noisy Healthcare Data	Intensive care medicine; Intensive care medicine; Intensive care medicine	Non-deep machine learning	Clinical data	Model specification and learning
T. Asvestopoulou, et al. (2019)	Towards a robust and accurate screening tool for dyslexia with data augmentation using GANs	Neurology	Non-deep machine learning; Other	Other	Input perturbations and alterations
T. P. Karnowski, et al. (2011)	Automatic Detection of Retina Disease: Robustness to Image Quality and Localization of Anatomy Structure	Opthalmology	Hybrid model	Image derived features	Input perturbations and alterations
Hong Hu, et al. (2008)	Robustness analysis of diversified ensemble decision tree algorithms for Microarray data classification	Hematology; Gastroenterology; Pulmonology; Other	Non-deep machine learning	Omics	Input perturbations and alterations
Nestor, Bret, et al. (2019)	Feature Robustness in Non-stationary Health Records: Caveats to Deployable Model Performance in Common Clinical Machine Learning Tasks	Intensive care medicine; Intensive cancer medicine	Linear regression model; Non-deep machine learning; Deep learning	Clinical data	Feature extraction and selection; External data and domain shift
Yip, Stephen SF, et al. (2020)	Performance and Robustness of Machine Learning-based Radiomic COVID-19 Severity Prediction	Pulmonology	Linear regression model	Image derived features	Label noise
Yilmaz, Ibrahim, et al. (2021)	On the Assessment of Robustness of Telemedicine Applications against Adversarial Machine Learning Attacks	Gynaecology	Deep learning	Image	Adversarial attacks

Kuijs, Merel, et al. (2021)	Interpretability Aware Model Training to Improve Robustness against Out-of-Distribution Magnetic Resonance Images in Alzheimer's Disease Classification	Neurology	Deep learning	Image	External data and domain shift
Oala, Luis, et al. (2020)	ML4H Auditing: From Paper to Practice	Opthalmology; Neurology; Hematology	Deep learning; Other; Non-deep machine learning; Deep learning	Image, Multimodal, Image	Input perturbations and alterations
Hsu, Yi-Te, et al. (2018)	Robustness against the channel effect in pathological voice detection	ORL	Deep learning	Other	External data and domain shift
Abdelhack, Mohamed, et al. (2023)	A Modulation Layer to Increase Neural Network Robustness Against Data Quality Issues	Intensive care medicine; Urology; Cardiology; Gynaecology; Pulmonology	Deep learning	Clinical data, Image derived features, Clinical data	Missing data
Hooper, Sarah M, et al. (2020)	Assessing Robustness to Noise: Low-Cost Head CT Triage	Neurology	Deep learning	Image	Input perturbations and alterations
Ishii, Shotaro, et al. (2021)	A Comparative Analysis of Robustness to Noise in Machine Learning Classifiers	Endocrinology; Gynaecology	Non-deep machine learning; Deep learning	Clinical data, Image derived features	Input perturbations and alterations
Liang, Paul Pu, et al. (2021)	MULTIBENCH: Multiscale Benchmarks for Multimodal Representation Learning	Intensive care medicine	Deep learning	Multimodal	Input perturbations and alterations; Missing data
Yao, Huaxiu, et al. (2022)	Wild-Time: A Benchmark of in-the-Wild Distribution Shift over Time	Intensive care medicine	Deep learning	Clinical data	External data and domain shift
Kulkarni, Mihir, et al. (2022)	Predicting Treatment Adherence of Tuberculosis Patients at Scale	Infectious diseases	Non-deep machine learning	Other	External data and domain shift; Model specification and learning
Lopez-Martinez, Daniel, et al. (2022)	Instability in clinical risk stratification models using deep learning	Other	Linear regression model; Deep learning	Clinical data	Model specification and learning
Saab, Khaled, et al. (2022)	Reducing Reliance on Spurious Features in Medical Image Classification with Spatial Specificity	Pulmonology; Dermatology	Deep learning	Image, Image	External data and domain shift
Zhu, Jiacheng, et al. (2022)	GeoECG: Data Augmentation via Wasserstein Geodesic Perturbation for Robust Electrocardiogram Prediction	Cardiology	Deep learning	Physiological signal	Adversarial attacks
Filos, Angelos, et al. (2019)	A Systematic Comparison of Bayesian Deep Learning Robustness in Diabetic Retinopathy Tasks	Opthalmology	Deep learning	Image	External data and domain shift
Taghanaki, Saeid Asgari, et al. (2018)	Vulnerability Analysis of Chest X-Ray Image Classification Against Adversarial Attacks	Pulmonology	Deep learning	Image	Adversarial attacks

Drukker, Karen, et al. (2005)	Robustness of computerized lesion detection and classification scheme across different breast US platforms	Gynaecology	Deep learning	Image derived features	External data and domain shift
Gruszauskas, Nicholas P., et al. (2008)	Performance of breast ultrasound computer-aided diagnosis: Dependence on image selection	Gynaecology	Deep learning	Image derived features	Feature extraction and selection; Model specification and learning
Ho WH, et al. (2021)	Artificial intelligence classification model for macular degeneration images: a robust optimization framework for residual neural networks.	Opthalmology	Deep learning	Image	Model specification and learning
Le EPV, et al. (2021)	Assessing robustness of carotid artery CT angiography radiomics in the identification of culprit lesions in cerebrovascular events.	Cardiology	Linear regression model	Image derived features	Feature extraction and selection
Gao M, et al. (2022)	Bayesian statistics-guided label refurbishment mechanism: Mitigating label noise in medical image classification.	Opthalmology; Opthalmology	Deep learning	Image, Image	Label noise
Ren LR, et al. (2020)	Correntropy induced loss based sparse robust graph regularized extreme learning machine for cancer classification.	Other	Deep learning	Omics	Input perturbations and alterations
Paschali, M, et al. (2018)	Generalizability vs. Robustness: Investigating Medical Imaging Networks Using Adversarial Examples	Dermatology	Deep learning	Image	Adversarial attacks
Ju, L, et al. (2022)	Improving Medical Images Classification With Label Noise Using Dual-Uncertainty Estimation	Dermatology; Urology; Opthalmology	Deep learning	Image, Image, Image	Label noise
Chen, Y, et al. (2021)	LDNNET: Towards Robust Classification of Lung Nodule and Cancer Using Lung Dense Neural Network	Pulmonology; Pulmonology	Deep learning	Image	Model specification and learning
Roland, Theresa, et al. (2022)	Machine learning based COVID-19 diagnosis from blood tests with robustness to domain shifts	Pulmonology; Pulmonology	Deep learning; Linear regression model; Non-deep machine learning	Clinical data	External data and domain shift
Shen C, et al. (2020)	On the Robustness of Deep Learning-based Lung-Nodule Classification for CT images with respect to image noise.	Pulmonology	Deep learning	Image	Input perturbations and alterations; Adversarial attacks; Model specification and learning
Anisetti, Marco, et al. (2023)	On the Robustness of Random Forest Against Untargeted Data Poisoning: An Ensemble-Based Approach	Opthalmology	Non-deep machine learning	Image derived features	Input perturbations and alterations; Label noise
Dong, MS, et al. (2021)	Preoperatively Estimating the Malignant Potential of Mediastinal Lymph Nodes: A Pilot Study Toward Establishing a Robust Radiomics Model Based on Contrast-Enhanced CT Imaging	Pulmonology	Linear regression model	Image derived features	External data and domain shift
Yun J, et al. (2019)	Radiomic features and multilayer perceptron network classifier: a robust MRI classification strategy for distinguishing glioblastoma from primary central nervous system lymphoma.	Neuro-oncology	Deep learning; Linear regression	Image derived	External data and domain shift

			model; Non-deep machine learning; Deep learning	features, Image	
Zhang, HX, et al. (2022)	Re-thinking and Re-labeling LID C-IDRI for Robust Pulmonary Cancer Prediction	Pulmonology	Deep learning	Image	Label noise
Moen, T, et al. (2018)	Robustness of Textural Features to Predict Stone Fragility Across Computed Tomography Acquisition and Reconstruction Parameters	Urology	Linear regression model	Image derived features	External data and domain shift
Singla M, et al. (2021)	pin¯ -TSVM: A Robust Transductive Support Vector Machine and its Application to the Detection of COVID-19 Infected Patients.	Pulmonology	Hybrid model	Image derived features	Label noise

Supplementary Table 2. List of data, model type, and medical applications classified as “Other”.

Domain extracted	List
Data type	Vocal/speech recording based measurements, clinical notes (discharge summaries), smartwatches and smartphones extracted features, topographic indices from placido ring images, gait data
Model type	Naive Bayes, custom model, linear discriminant analysis, quadratic discriminant analysis, partial least squares-discriminant analysis, gaussian process, non-negative least squares (NNLS), graph-based semi-supervised learning
Medical application	Lymphoma detection, lipomatous soft-tissue tumours classification, diabetic foot ulcer classification, wound classification, cancer classification, cancer patient survival, computational pathology analysis, metastatic cancer detection, sarcoidosis detection, rheumatoid arthritis disease prediction, prediction of outpatient deterioration causing hospitalization

Supplementary Table 3. Different sources of variations encountered in healthcare.

Measure	Concept(s) tackled
Patient motion	Artefacts caused by patient movement during data acquisition, such as imaging or ECG recording, leading to distorted data.
Noise from acquisition devices	Artefacts originating from the devices used to collect data, such as imaging machines or sensors, which can degrade data quality.
Environmental noise	Variations introduced by the environment in which data is acquired, such as background noise or lighting conditions.
Measurement noise	Noise in lab exams measurement.
Image alterations (e.g., rotation, brightness)	Changes made to images during pre-processing, such as rotating or adjusting brightness.
Image compression and transmission	Loss of data quality due to compression and transmission, which can introduce artefacts and affect model accuracy.
Missing completely at random	Missing data that occurs without any specific pattern, unrelated to any other measured variables.
Missing due to clinical reasons	Missing data that occurs due to an underlying clinical reason, such as a test not being ordered based on a patient's health condition.
Medical uncertainty	Mismatch between the label used to train the model and the true diagnosis caused by annotators uncertainty.
Proxy labelling	Discrepancies arising from using surrogate outcomes instead of direct measures of the target variable.
Automatic labelling tools	Errors introduced by using automated tools to generate labels for training data.
Spatial heterogeneity	Differences in labels across different regions or part of an input.
Rare diseases	Underrepresentation of certain conditions, leading to limited data for training and validating models.
Feature selection methods	Variations stemming from different methods used to select features for model training.
Features extraction under different settings	Differences arising from the methods or conditions under which features are extracted from data.
Model selection procedure	Variations due to different methods used to select the best model from a set of candidates.
Model parametrisation	Variations due to different choices of model parameters.
Training hyperparameters	Variations due to different choices of training hyperparameters, such as learning rate or batch size.
Model evaluation method	Variations due to the use of different evaluation metrics or validation methods.
Uncertainty in model predictions	Variability in model output (predicted probabilities).
Model explainability	Variations in methods used to explain model decisions.
Different healthcare system	Variability in data and outcomes due to differences in healthcare systems across regions or countries.
Different care settings	Variability in different care settings.
Acquisition devices/platforms/protocols	Differences due to variations in data acquisition devices, platforms, or protocols.

Supplementary Table 4. List of methods used to evaluate the robustness of machine learning models.

Measure	Concept(s) tackled	References
Measure of the percentage of altered samples correctly classified given an acceptable accuracy	Input alterations and perturbations	1–3
Measure of model performance on altered samples	Input alterations and perturbations, Adversarial attacks, Missing data	4–7
Measures by how much on average the model's output probability changes after the sample is altered	Input alterations and perturbations	8
Measure of the relative errors between model performance on clean and altered samples	Input alterations and perturbations, Label noise, Missing data	4,5,7,9-12
Measure of the relative errors between model trained on clean samples and a model trained on altered samples	Label noise, Input alterations and perturbations, Missing data	13
Measures mismatch between degree of alteration and human intuitions	Input alterations and perturbations	4
Measure of the number of time a model changes its prediction after a sample has been altered several times	Input alterations and perturbations	14,15
Measure of the percentage of samples for which the model changes its predictions after alteration	Input alterations and perturbations, Adversarial attacks	5,8,15–20
Measure of the confidence score/uncertainty/intervals of model predictions	Input alterations and perturbations, Adversarial attacks, Model specification and learning, External data and domain shift	21–25
Measure of the degree of alteration of a sample	Adversarial attacks	16
Measure of similarity between clean and altered samples	Adversarial attacks	16
Measure of model performance tradeoff between clean sample and altered samples	Adversarial attacks	26
Measures the stability based on the average model performance on different runs (e.g. with different hyperparameters, random seeds, etc.)	Model specification and learning, External data and domain shift	27-30
Measure of the number of samples for which the predictions change if another model with similar performance was used.	Model specification and learning	31
Measure of the reliability of extracted features under different conditions	Feature selection and extraction, External data and domain shift	32-42
Measure of the similarity between two (or more) sets of features obtained on resampled datasets/disjoints partition of the data	Feature selection and extraction	43-46
Compares model performance using data extracted under different conditions/or using different preprocessing steps	Feature selection and extraction	40
Measure of the ability to recover noisy labels	Label noise	47
Measure of the average sensitivity for each class	Imbalanced data	48
Measure of the concordance of different explanation methods for the same samples	Model specification and learning	49
Measures whether similar inputs have similar explanations	Model specification and learning	50
Measures model performance on samples without signs of spurious correlations	External data and domain shift	51
Measures model performance on samples from future time period	External data and domain shift	52
Measures the most correlated attributes with model predictions	External data and domain shift	53

Supplementary Table 5. Concepts of robustness stratified by medical specialty.

Medical specialty	Input perturbations and alterations	Label noise	Missing data	Imbalanced data	Feature selection and reliability	Model parametrization and learning	External data and domain shift	Adversarial attacks
Pulmonology	17 (12.0%)	12 (21.1%)	2 (9.5%)	5 (33.3%)	10 (16.7%)	9 (11.1%)	18 (16.4%)	13 (32.5%)
Gynaecology	21 (14.8%)	10 (17.5%)	7 (33.3%)	0 (0.0%)	8 (13.3%)	22 (27.2%)	15 (13.6%)	2 (5.0%)
Neurology	29 (20.4%)	4 (7.0%)	0 (0.0%)	2 (13.3%)	5 (8.3%)	10 (12.3%)	23 (20.9%)	0 (0.0%)
Dermatology	10 (7.0%)	8 (14.0%)	0 (0.0%)	1 (6.7%)	1 (1.7%)	1 (1.2%)	5 (4.5%)	7 (17.5%)
Gastroenterology	8 (5.6%)	2 (3.5%)	1 (4.8%)	1 (6.7%)	6 (10.0%)	7 (8.6%)	5 (4.5%)	2 (5.0%)
Intensive care	2 (1.4%)	0 (0.0%)	5 (23.8%)	0 (0.0%)	6 (10.0%)	5 (6.2%)	13 (11.8%)	1 (2.5%)
Ophthalmology	9 (6.3%)	5 (8.8%)	0 (0.0%)	0 (0.0%)	0 (0.0%)	4 (4.9%)	5 (4.5%)	6 (15.0%)
Cardiology	10 (7.0%)	2 (3.5%)	1 (4.8%)	0 (0.0%)	1 (1.7%)	4 (4.9%)	2 (1.8%)	3 (7.5%)
Urology	7 (4.9%)	3 (5.3%)	1 (4.8%)	0 (0.0%)	3 (5.0%)	2 (2.5%)	5 (4.5%)	1 (2.5%)
Haematology	8 (5.6%)	1 (1.8%)	0 (0.0%)	0 (0.0%)	4 (6.7%)	8 (9.9%)	0 (0.0%)	0 (0.0%)
Neuro-oncology	1 (0.7%)	0 (0.0%)	0 (0.0%)	0 (0.0%)	10 (16.7%)	3 (3.7%)	5 (4.5%)	1 (2.5%)
Psychiatry	3 (2.1%)	3 (5.3%)	4 (19.0%)	3 (20.0%)	0 (0.0%)	0 (0.0%)	2 (1.8%)	0 (0.0%)
Infectious diseases	1 (0.7%)	1 (1.8%)	0 (0.0%)	1 (6.7%)	0 (0.0%)	1 (1.2%)	5 (4.5%)	2 (5.0%)
Endocrinology	5 (3.5%)	2 (3.5%)	0 (0.0%)	1 (6.7%)	0 (0.0%)	0 (0.0%)	1 (0.9%)	0 (0.0%)
ENT	2 (1.4%)	0 (0.0%)	0 (0.0%)	0 (0.0%)	0 (0.0%)	2 (2.5%)	3 (2.7%)	0 (0.0%)
Other	9 (6.3%)	4 (7.0%)	0 (0.0%)	1 (6.7%)	6 (10.0%)	3 (3.7%)	3 (2.7%)	2 (5.0%)

ENT: Ear-nose-throat

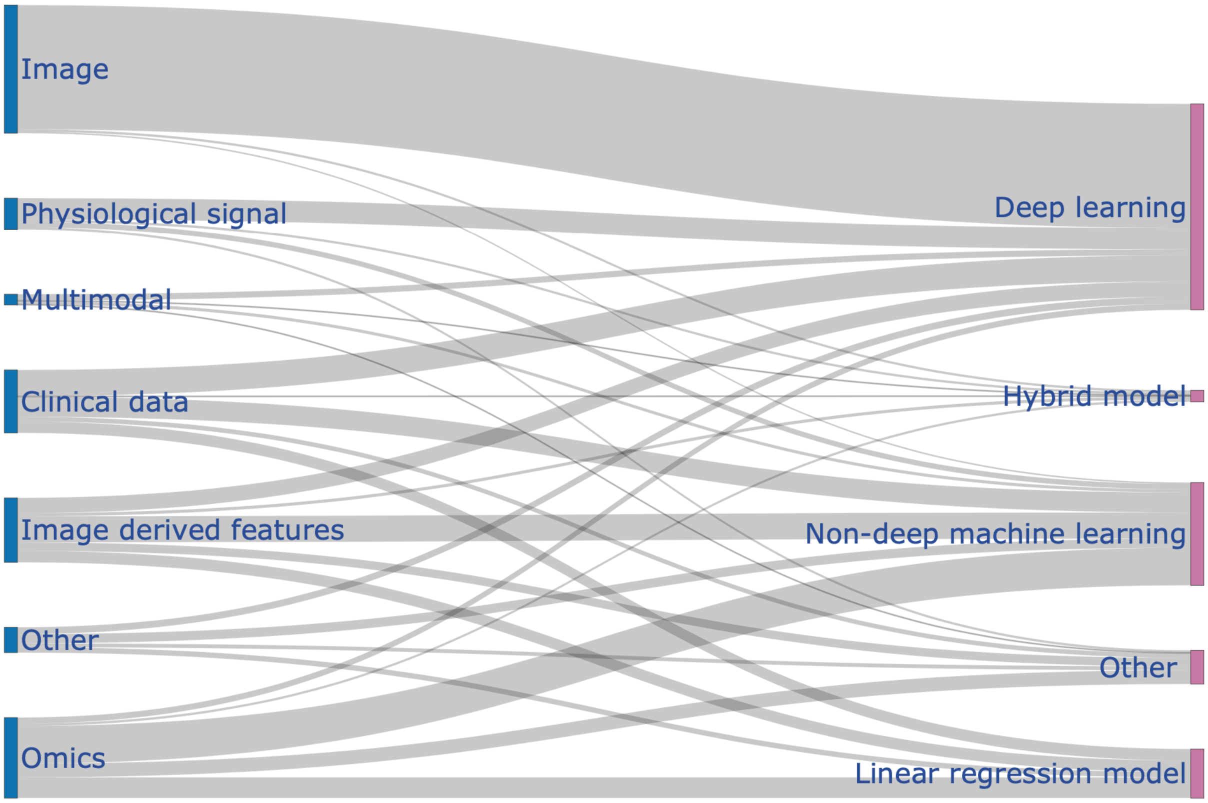

Supplementary Figure 1. Alluvial diagram of data types and predictive model types.

The alluvial diagram illustrates the associations between data types and the predictive models used. The thickness of each path represents the frequency of corresponding data-model pairings across the included studies.

Supplementary Table 6. Preferred Reporting Items for Systematic reviews and Meta-Analyses extension for Scoping Reviews (PRISMA-ScR) Checklist

SECTION	ITEM	PRISMA-ScR CHECKLIST ITEM	REPORTED ON PAGE #
**TITLE**			
Title	1	Identify the report as a scoping review.	1
**ABSTRACT**			
Structured summary	2	Provide a structured summary that includes (as applicable): background, objectives, eligibility criteria, sources of evidence, charting methods, results, and conclusions that relate to the review questions and objectives.	2
**INTRODUCTION**			
Rationale	3	Describe the rationale for the review in the context of what is already known. Explain why the review questions/objectives lend themselves to a scoping review approach.	3-4
Objectives	4	Provide an explicit statement of the questions and objectives being addressed with reference to their key elements (e.g., population or participants, concepts, and context) or other relevant key elements used to conceptualize the review questions and/or objectives.	4
**METHODS**			
Protocol and registration	5	Indicate whether a review protocol exists; state if and where it can be accessed (e.g., a Web address); and if available, provide registration information, including the registration number.	13
Eligibility criteria	6	Specify characteristics of the sources of evidence used as eligibility criteria (e.g., years considered, language, and publication status), and provide a rationale.	13
Information sources*	7	Describe all information sources in the search (e.g., databases with dates of coverage and contact with authors to identify additional sources), as well as the date the most recent search was executed.	13
Search	8	Present the full electronic search strategy for at least 1 database, including any limits used, such that it could be repeated.	Supplementary Tables 7-9
Selection of sources of evidence†	9	State the process for selecting sources of evidence (i.e., screening and eligibility) included in the scoping review.	13-14
Data charting process‡	10	Describe the methods of charting data from the included sources of evidence (e.g., calibrated forms or forms that have been tested by the team before their use, and whether data charting was done independently or in duplicate) and any processes for obtaining and confirming data from investigators.	14
Data items	11	List and define all variables for which data were sought and any assumptions and simplifications made.	14
Critical appraisal of individual sources of evidence§	12	If done, provide a rationale for conducting a critical appraisal of included sources of evidence; describe the methods used and how this information was used in any data synthesis (if appropriate).	N.A
Synthesis of results	13	Describe the methods of handling and summarizing the data that were charted.	14
**RESULTS**			
Selection of sources of evidence	14	Give numbers of sources of evidence screened, assessed for eligibility, and included in the review, with reasons for exclusions at each stage, ideally using a flow diagram.	Figure 1

SECTION	ITEM	PRISMA-ScR CHECKLIST ITEM	REPORTED ON PAGE #
Characteristics of sources of evidence	15	For each source of evidence, present characteristics for which data were charted and provide the citations.	Table 1
Critical appraisal within sources of evidence	16	If done, present data on critical appraisal of included sources of evidence (see item 12).	N.A
Results of individual sources of evidence	17	For each included source of evidence, present the relevant data that were charted that relate to the review questions and objectives.	Supplementary Table 1
Synthesis of results	18	Summarize and/or present the charting results as they relate to the review questions and objectives.	Table 2, Figures 2-3, Supplementary Tables 2-5, Supplementary Figure 1
**DISCUSSION**			
Summary of evidence	19	Summarize the main results (including an overview of concepts, themes, and types of evidence available), link to the review questions and objectives, and consider the relevance to key groups.	4-7, 10-11
Limitations	20	Discuss the limitations of the scoping review process.	11-12
Conclusions	21	Provide a general interpretation of the results with respect to the review questions and objectives, as well as potential implications and/or next steps.	10-12
**FUNDING**			
Funding	22	Describe sources of funding for the included sources of evidence, as well as sources of funding for the scoping review. Describe the role of the funders of the scoping review.	15

JBI = Joanna Briggs Institute; PRISMA-ScR = Preferred Reporting Items for Systematic reviews and Meta-Analyses extension for Scoping Reviews.

* Where *sources of evidence* (see second footnote) are compiled from, such as bibliographic databases, social media platforms, and Web sites.

† A more inclusive/heterogeneous term used to account for the different types of evidence or data sources (e.g., quantitative and/or qualitative research, expert opinion, and policy documents) that may be eligible in a scoping review as opposed to only studies. This is not to be confused with *information sources* (see first footnote).

‡ The frameworks by Arksey and O'Malley (6) and Levac and colleagues (7) and the JBI guidance (4, 5) refer to the process of data extraction in a scoping review as data charting.

§ The process of systematically examining research evidence to assess its validity, results, and relevance before using it to inform a decision. This term is used for items 12 and 19 instead of "risk of bias" (which is more applicable to systematic reviews of interventions) to include and acknowledge the various sources of evidence that may be used in a scoping review (e.g., quantitative and/or qualitative research, expert opinion, and policy document).

From: Tricco AC, Lillie E, Zarin W, O'Brien KK, Colquhoun H, Levac D, et al. PRISMA Extension for Scoping Reviews (PRISMAScR): Checklist and Explanation. Ann Intern Med. 2018;169:467–473. doi: 10.7326/M18-0850.

Supplementary Table 7. Search strategy on PubMed.

#1	("Machine Learning"[MeSH Terms] OR "Artificial Intelligence"[MeSH Terms:noexp] OR "Machine Learning"[Title/Abstract] OR "deep learning"[Title/Abstract] OR "Artificial Intelligence"[Title/Abstract] OR "statistical learning"[Title/Abstract] OR "computer vision"[Title/Abstract] OR "prediction model"[Title/Abstract] OR "neural network"[Title/Abstract])
#2	"robust*"[Title] OR "perturbation*"[Title] OR "nois*"[Title]
#3	#1 AND #2

Supplementary Table 8. Search strategy on IEEE Xplore.

(((("All Metadata":artificial intelligence OR "All Metadata":deep learning OR "All Metadata":machine learning OR "All Metadata":prediction model OR "All Metadata":statistical learning OR "All Metadata":computer vision OR "All Metadata":neural network)<br>AND ("Document Title":"robust*" OR "Document Title":"perturbation*" OR "Document Title":"nois*")<br>AND ("All Metadata":"health*" OR "All Metadata":"clinic*" OR "All Metadata":"medic*" OR "All Metadata":"diagnos*" OR "All Metadata":"prognos*" OR "All Metadata":"hospital" OR "All Metadata":"hospitals" OR "All Metadata":"patient" OR "All Metadata":"patients"))))

Supplementary Table 9. Search strategy on Web of Science.

#1	TS=(machine learning) OR TS=(artificial intelligence) OR TS=(deep learning) OR TS=(statistical learning) OR TS=(computer vision) OR TS=(prediction model) OR TS=(neural network)
#2	TI=(robust*) OR TI=(perturbation*) OR TI=(nois*)
#3	TS=(health*) OR TS=(clinic*) OR TS=(medic*) OR TS=(diagnos*) OR TS=(prognos*) OR TS=(hospital*) OR TS=(patient*)
#4	#1 AND #2 AND #3

Supplementary References

1. Arcaini, P., Bombarda, A., Bonfanti, S. & Gargantini, A. Dealing with Robustness of Convolutional Neural Networks for Image Classification. in *2020 IEEE International Conference On Artificial Intelligence Testing (AITest)* 7–14 (IEEE, Oxford, UK, 2020). doi:10.1109/AITEST49225.2020.00009.
2. Arcaini, P., Bombarda, A., Bonfanti, S. & Gargantini, A. Efficient Computation of Robustness of Convolutional Neural Networks. in *2021 IEEE International Conference on Artificial Intelligence Testing (AITest)* 21–28 (2021). doi:10.1109/AITEST52744.2021.00015.
3. Arcaini, P., Bombarda, A., Bonfanti, S. & Gargantini, A. ROBY: a Tool for Robustness Analysis of Neural Network Classifiers. in *2021 14th IEEE Conference on Software Testing, Verification and Validation (ICST)* 442–447 (2021). doi:10.1109/ICST49551.2021.00057.
4. Zhang, Y. *et al.* Benchmarking the Robustness of Deep Neural Networks to Common Corruptions in Digital Pathology. in *Medical Image Computing and Computer Assisted Intervention – MICCAI 2022* (eds. Wang, L., Dou, Q., Fletcher, P. T., Speidel, S. & Li, S.) 242–252 (Springer Nature Switzerland, Cham, 2022). doi:10.1007/978-3-031-16434-7_24.
5. Maron, R. C. *et al.* A benchmark for neural network robustness in skin cancer classification. *Eur. J. Cancer Oxf. Engl. 1990* **155**, 191–199 (2021).
6. Roh, J. Impact of Adversarial Training on the Robustness of Deep Neural Networks. in *2022 IEEE 5th International Conference on Information Systems and Computer Aided Education (ICISCAE)* 560–566 (2022). doi:10.1109/ICISCAE55891.2022.9927611.
7. Liang, P. P. *et al.* MULTIBENCH: Multiscale Benchmarks for Multimodal Representation Learning.
8. Maron, R. C. *et al.* Robustness of convolutional neural networks in recognition of pigmented skin lesions. *Eur. J. Cancer Oxf. Engl. 1990* **145**, 81–91 (2021).
9. Shanthini, A., Vinodhini, G., Chandrasekaran, R. M. & Supraja, P. A taxonomy on impact of label noise and feature noise using machine learning techniques. *Soft Comput.* **23**, 8597–8607 (2019).
10. Ishii, S. & Ljunggren, D. *A Comparative Analysis of Robustness to Noise in Machine Learning Classifiers*. (2021).
11. Oala, L. *et al.* ML4H Auditing: From Paper to Practice. in (2020).
12. Robust Skin Disease Classification by Distilling Deep Neural Network Ensemble for the Mobile Diagnosis of Herpes Zoster | IEEE Journals & Magazine | IEEE Xplore. https://ieeexplore.ieee.org/document/9335582.

13. Anisetti, M. *et al.* On the Robustness of Random Forest Against Untargeted Data Poisoning: An Ensemble-Based Approach. *IEEE Trans. Sustain. Comput.* **8**, 540–554 (2023).

14. Gao, Y., Xiong, J., Shen, C. & Jia, X. Improving robustness of a deep learning-based lung-nodule classification model of CT images with respect to image noise. *Phys. Med. Biol.* **66**, (2021).

15. Shen, C. *et al.* On the robustness of deep learning-based lung-nodule classification for CT images with respect to image noise. *Phys. Med. Biol.* **65**, 245037 (2020).

16. Wang, K., Wang, G., Chen, N. & Chen, T. How Robust is Your Automatic Diagnosis Model? in *2019 IEEE International Conference on Bioinformatics and Biomedicine (BIBM)* 877–884 (2019). doi:10.1109/BIBM47256.2019.8983217.

17. Xu, M., Zhang, T., Li, Z., Liu, M. & Zhang, D. Towards evaluating the robustness of deep diagnostic models by adversarial attack. *Med. Image Anal.* **69**, 101977 (2021).

18. Hoque, M. A., Haque, S., Debnath, S. K. & Ahiduzzaman, Md. Investigating the Robustness of Deep Neural Network Based COVID-19 Detection Models Against Universal Adversarial Attacks. in *2021 3rd International Conference on Sustainable Technologies for Industry 4.0 (STI)* 1–6 (2021). doi:10.1109/STI53101.2021.9732570.

19. Allyn, J., Allou, N., Vidal, C., Renou, A. & Ferdynus, C. Adversarial attack on deep learning-based dermatoscopic image recognition systems: Risk of misdiagnosis due to undetectable image perturbations. *Medicine (Baltimore)* **99**, e23568 (2020).

20. Ghaffari Laleh, N. *et al.* Adversarial attacks and adversarial robustness in computational pathology. *Nat. Commun.* **13**, 5711 (2022).

21. Xu, M., Zhang, T. & Zhang, D. MedRDF: A Robust and Retrain-Less Diagnostic Framework for Medical Pretrained Models Against Adversarial Attack. *IEEE Trans. Med. Imaging* **41**, 2130–2143 (2022).

22. Jaskari, J. *et al.* Uncertainty-Aware Deep Learning Methods for Robust Diabetic Retinopathy Classification. *IEEE Access* **10**, 76669–76681 (2022).

23. Soukup, M., Cho, H. & Lee, J. K. Robust classification modeling on microarray data using misclassification penalized posterior. *Bioinforma. Oxf. Engl.* **21 Suppl 1**, i423-430 (2005).

24. Abbas, M. R. *et al.* Accuracy Rejection Normalized-Cost Curves (ARNCCs): A Novel 3-Dimensional Framework for Robust Classification. *IEEE Access* **7**, 160125–160143 (2019).

25. Filos, A. *et al.* A Systematic Comparison of Bayesian Deep Learning Robustness in Diabetic Retinopathy Tasks. Preprint at https://doi.org/10.48550/arXiv.1912.10481 (2019).

26. Ma, L. & Liang, L. A regularization method to improve adversarial robustness of neural networks for ECG signal classification. *Comput. Biol. Med.* **144**, 105345 (2022).

27. Feng, X., Zhang, S., Xu, L., Huang, X. & Chen, Y. Robust Classification Model for Diabetic Retinopathy Based on the Contrastive Learning Method with a Convolutional Neural Network. *Appl. Sci.* **12**, 12071 (2022).

28. Ho, W.-H. *et al.* Artificial intelligence classification model for macular degeneration images: a robust optimization framework for residual neural networks. *BMC Bioinformatics* **22**, 148 (2021).

29. Yun, J. *et al.* Radiomic features and multilayer perceptron network classifier: a robust MRI classification strategy for distinguishing glioblastoma from primary central nervous system lymphoma. *Sci. Rep.* **9**, 5746 (2019).

30. Lopez-Martinez, D. *et al.* Instability in clinical risk stratification models using deep learning. in *Proceedings of the 2nd Machine Learning for Health symposium* 552–565 (PMLR, 2022).

31. Kulkarni, M. *et al.* Predicting Treatment Adherence of Tuberculosis Patients at Scale. Preprint at https://doi.org/10.48550/arXiv.2211.02943 (2022).

32. Le, E. P. V. *et al.* Assessing robustness of carotid artery CT angiography radiomics in the identification of culprit lesions in cerebrovascular events. *Sci. Rep.* **11**, 3499 (2021).

33. Suter, Y. *et al.* Radiomics for glioblastoma survival analysis in pre-operative MRI: exploring feature robustness, class boundaries, and machine learning techniques. *Cancer Imaging* **20**, 55 (2020).

34. Sudjai, N. *et al.* Robustness of Radiomic Features: Two-Dimensional versus Three-Dimensional MRI-Based Feature Reproducibility in Lipomatous Soft-Tissue Tumors. *Diagn. Basel Switz.* **13**, 258 (2023).

35. Könik, A., Miskin, N., Guo, Y., Shinagare, A. B. & Qin, L. Robustness and performance of radiomic features in diagnosing cystic renal masses. *Abdom. Radiol. N. Y.* **46**, 5260–5267 (2021).

36. Poirot, M. G. *et al.* Robustness of radiomics to variations in segmentation methods in multimodal brain MRI. *Sci. Rep.* **12**, 16712 (2022).

37. Oliveira, C. *et al.* Preselection of robust radiomic features does not improve outcome modelling in non-small cell lung cancer based on clinical routine FDG-PET imaging. *EJNMMI Res.* **11**, 79 (2021).

38. Ren, Q. *et al.* Assessing the robustness of radiomics/deep learning approach in the identification of efficacy of anti-PD-1 treatment in advanced or metastatic non-small cell lung carcinoma patients. *Front. Oncol.* **12**, 952749 (2022).

39. Ubaldi, L., Saponaro, S., Giuliano, A., Talamonti, C. & Retico, A. Deriving quantitative information from multiparametric MRI via Radiomics: Evaluation of the robustness and predictive value of radiomic features in the discrimination of low-grade versus high-grade gliomas with machine learning. *Phys. Medica PM Int. J. Devoted Appl. Phys. Med. Biol. Off. J. Ital. Assoc. Biomed. Phys. AIFB* **107**, 102538 (2023).

40. Chong, D. Y. *et al.* Robustness-Driven Feature Selection in Classification of Fibrotic Interstitial Lung Disease Patterns in Computed Tomography Using 3D Texture Features. *IEEE Trans. Med. Imaging* **35**, 144–157 (2016).

41. Karagöz, A. & Guvenis, A. Robust whole-tumour 3D volumetric CT-based radiomics approach for predicting the WHO/ISUP grade of a ccRCC tumour. *Comput. Methods Biomech. Biomed. Eng. Imaging Vis.* **11**, 665–677 (2023).

42. Dong, M. *et al.* Preoperatively Estimating the Malignant Potential of Mediastinal Lymph Nodes: A Pilot Study Toward Establishing a Robust Radiomics Model Based on Contrast-Enhanced CT Imaging. *Front. Oncol.* **10**, 558428 (2020).

43. Yassi, M. & Moattar, M. H. Robust and stable feature selection by integrating ranking methods and wrapper technique in genetic data classification. *Biochem. Biophys. Res. Commun.* **446**, 850–856 (2014).

44. Mi, H., Petitjean, C., Dubray, B., Vera, P. & Ruan, S. Robust feature selection to predict tumor treatment outcome. *Artif. Intell. Med.* **64**, 195–204 (2015).

45. Adnan, N., Najnin, T. & Ruan, J. A Robust Personalized Classification Method for Breast Cancer Metastasis Prediction. *Cancers* **14**, 5327 (2022).

46. Venkatesh, R., Rowland, C., Huang, H., Abar, O. T. & Sninsky, J. Robust Model Selection Using Cross Validation: A Simple Iterative Technique for Developing Robust Gene Signatures in Biomedical Genomics Applications. in *2006 5th International Conference on Machine Learning and Applications (ICMLA'06)* 193–198 (2006). doi:10.1109/ICMLA.2006.45.

47. Ying, X., Liu, H. & Huang, R. COVID-19 chest X-ray image classification in the presence of noisy labels. *Displays* **77**, 102370 (2023).

48. Foahom Gouabou, A. C., Iguernaissi, R., Damoiseaux, J.-L., Moudafi, A. & Merad, D. End-to-End Decoupled Training: A Robust Deep Learning Method for Long-Tailed Classification of Dermoscopic Images for Skin Lesion Classification. *Electronics* **11**, 3275 (2022).
49. Malafaia, M., Silva, F., Neves, I., Pereira, T. & Oliveira, H. P. Robustness Analysis of Deep Learning-Based Lung Cancer Classification Using Explainable Methods. *IEEE Access* **10**, 112731–112741 (2022).
50. Amador, T., Saturnino, S., Veloso, A. & Ziviani, N. Early identification of ICU patients at risk of complications: Regularization based on robustness and stability of explanations. *Artif. Intell. Med.* **128**, 102283 (2022).
51. Saab, K. *et al.* Reducing Reliance on Spurious Features in Medical Image Classification with Spatial Specificity. in *Proceedings of the 7th Machine Learning for Healthcare Conference* 760–784 (PMLR, 2022).
52. Yao, H. *et al.* Wild-Time: A Benchmark of in-the-Wild Distribution Shift over Time. *Adv. Neural Inf. Process. Syst.* **35**, 10309–10324 (2022).
53. O'Brien, M., Bukowski, J., Hager, G., Pezeshk, A. & Unberath, M. Evaluating neural network robustness for melanoma classification using mutual information. in *Medical Imaging 2022: Image Processing* vol. 12032 173–177 (SPIE, 2022).
